# Atg4B and Cathepsin B-Triggered in Situ Luciferin Formation for Precise Cancer Autophagy Bioluminescence Imaging

**DOI:** 10.1021/acscentsci.3c00696

**Published:** 2023-11-08

**Authors:** Xiaotong Cheng, Tiantian Xia, Xianbao Sun, Guowei Liang, Xiaoyang Liu, Gaolin Liang

**Affiliations:** State Key Laboratory of Digital Medical Engineering, School of Biological Science and Medical Engineering, Southeast University, Nanjing 210096, China

## Abstract

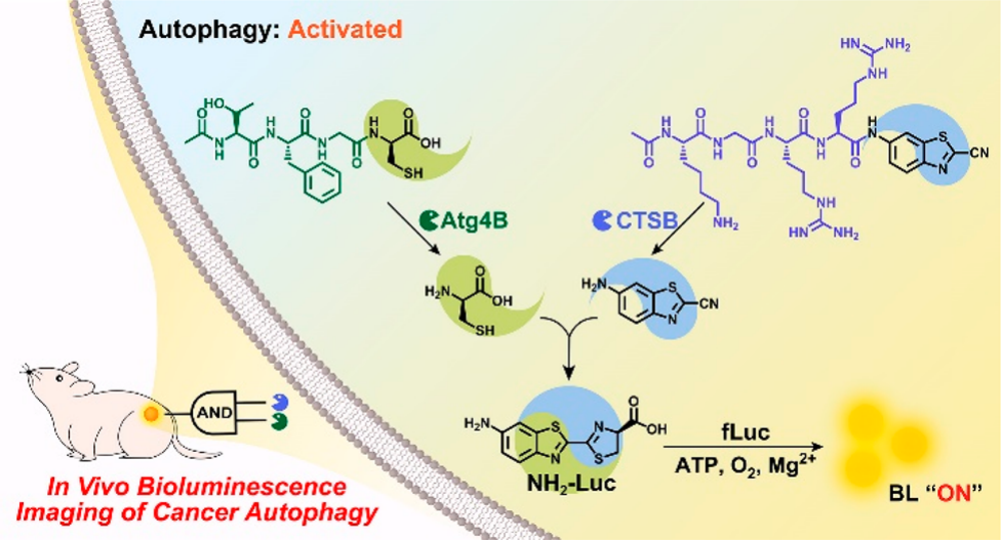

Autophagy plays a crucial role in tumorigenesis and progression, but current approaches to visualize it in vivo show limited precision due to their single-analyte-responsive mode. Hence, by simultaneously employing dual autophagy enzymes Atg4B and cathepsin B to trigger the in situ formation of luciferin, we herein propose a strategy for precise autophagy bioluminescence imaging. An Atg4B-responsive peptide Ac-Thr-Phe-Gly-d-Cys (TFGC) and a cathepsin B-activatable compound Ac-Lys-Gly-Arg-Arg-CBT (KGRR-CBT) were rationally designed. During tumor autophagy, these two compounds were uptaken by cancer cells and cleaved by their corresponding enzymes to yield d-cysteine and 2-cyano-6-aminobenzothiazole, respectively, which underwent a CBT-Cys click reaction to yield d-aminoluciferin, turning the bioluminescence “on”. The responsiveness of these two compounds toward the two enzymes was tested in vitro, and the ability to turn bioluminescence “on” was validated in living cancer cells and in vivo. We anticipate that our precise autophagy imaging strategy could be further applied for the diagnosis of autophagy-related diseases in the near future.

## Abstract 2

Intracellular Atg4B and cathepsin B-catalyzed d-luciferin formation was accomplished for precise bioluminescence imaging of cancer autophagy.

## Introduction

As a crucial lysosome-dependent metabolic process, autophagy clears microorganism invaders, misfolded proteins, and damaged (or useless) organelles to maintain homeostasis.^1,2^ Extensive studies corroborated that autophagy dysfunction was responsible for diverse diseases including neurodegenerative disorders (e.g., Alzheimer’s disease, transmissible spongiform encephalopathies, Parkinson’s disease, and Huntington’s disease),^1,3^ metabolic diseases,^4^ infectious diseases,^5^ aging,^6^ and cancers.^7^ Among these studies, cancer-associated autophagy has raised intensive interest. It closely overlaps with the tumorigenesis signaling pathways and plays a paradoxical role in cancer progression or treatment.^8^ On one hand, autophagy may help cancer cells to survive under hypoxia and metabolic stress conditions.^9^ On the other hand, it may inhibit tumor progression by degrading certain organelles or proteins for cell growth.^10^ Therefore, it is necessary and significant to visualize/assess autophagy at the subcellular level, which helps to reveal the underlying mechanisms and further benefits cancer therapy via rational autophagy regulation.

Now, to evaluate autophagy in tumors, transmission electron microscopy (TEM), genetic labeling, and Western blot are commonly employed as the first-line methods to detect autophagy hallmarks including autophagy-related subcellular structures, light chain 3 (LC3), Atg4, Beclin-1, and lysosomal associated membrane proteins (i.e., Lamp-1 and Lamp-2).^7,11^ However, most of these approaches are time-consuming, sampling demanding, and unable to be applied for real-time autophagy visualization in vivo. Some efforts have tried to address these issues by fabricating smart fluorescent autophagy nanoprobes, but they are designed for one-to-one sensing of the autophagy hallmarks.^12−14^ It can be envisaged that, could the probes (or nanoprobes) simultaneously sense dual (or more) autophagy analytes, it might significantly enhance the precision sensing/imaging of autophagy. However, such type of probes have remained to be developed.

**Scheme 1:**
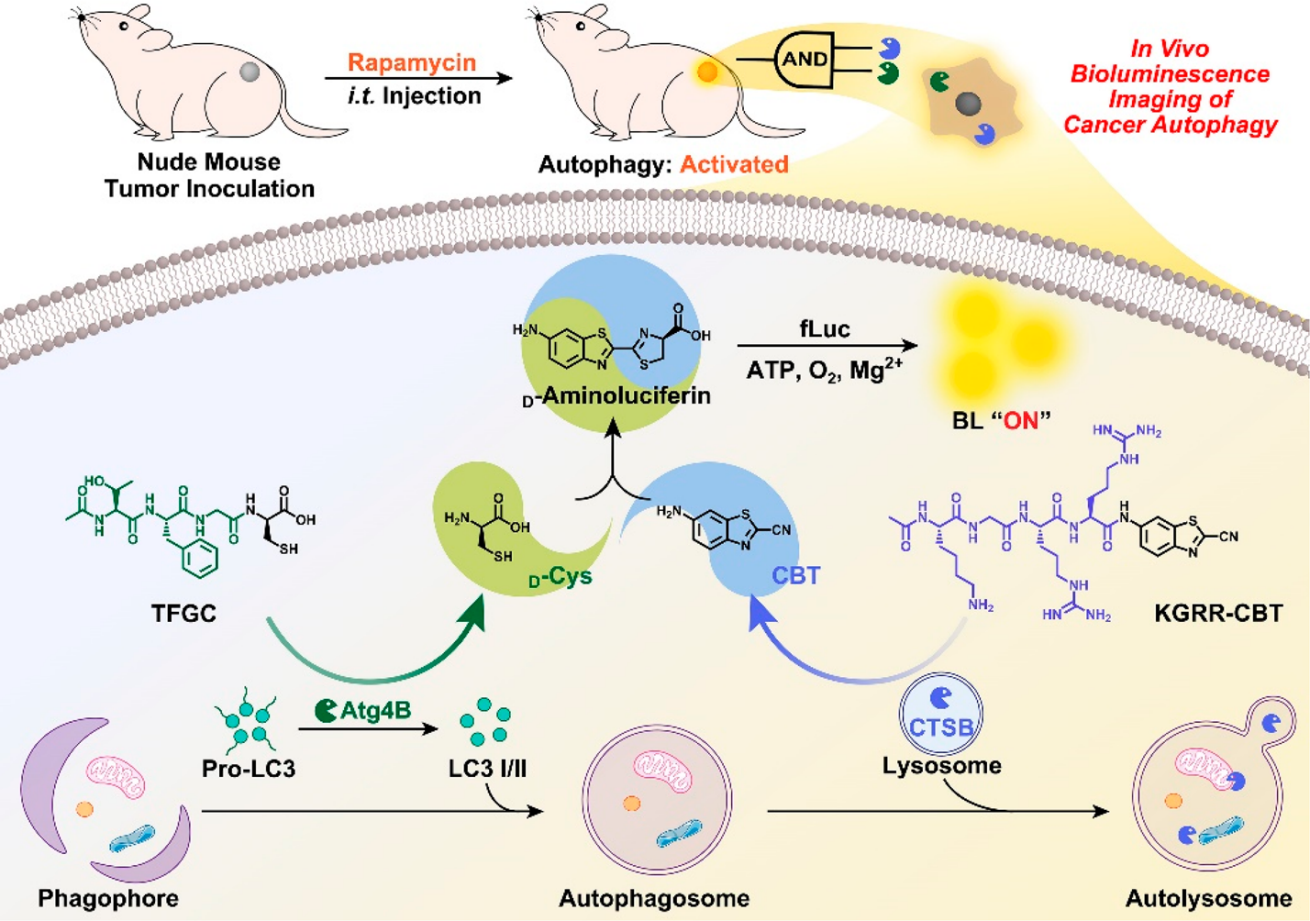
Schematic Illustration of Employing the Activated Autophagy Enzymes Atg4B and CTSB to Cleave TFGC and KGRR-CBT for in Situ Formation of d-Cys and CBT Intracellularly generated d-Cys and CBT undergo a CBT-Cys click reaction to yield d-aminoluciferin for precise cancer autophagy bioluminescence imaging.

Hence, by simultaneously employing dual autophagy enzymes to trigger the in situ formation of d-aminoluciferin, we herein proposed a “smart” strategy for precise autophagy bioluminescence (BL) imaging. Compared with fluorescence imaging, BL imaging does not need external light excitation, thus it circumvents the exogenous light-caused problems (e.g., photobleaching and autofluorescence^15,16^), conferring higher signal-to-noise ratios.^17^ As one of the most widely used BL systems,^16,18^ the d-aminoluciferin–luciferase pair was employed in this study to design two luciferin precursors for in vivo BL imaging of dual autophagy enzymes, namely, Atg4B (a crucial cysteine protease involved in LC3 processing and recycling for autophagosome biogenesis^19,20^) and cathepsin B (CTSB, an essential hydrolase in autolysosomes^21^), respectively. As shown in Scheme 1, the complementary luciferin precursors d-cysteine (d-Cys) and 2-cyano-6-aminobenzothiazole (CBT) were caged with the peptide substrates Ac-Thr-Phe-Gly (TFG) and Ac-Lys-Gly-Arg-Arg (KGRR) to afford an Atg4B-responsive peptide Ac-Thr-Phe-Gly-d-Cys (TFGC) and a CTSB-activatable compound Ac-Lys-Gly-Arg-Arg-CBT (KGRR-CBT), respectively. During tumor autophagy, these two compounds are cleaved by their corresponding autophagy enzymes to yield d-Cys and CBT, respectively, which undergo a CBT-Cys click reaction to yield d-aminoluciferin inside cancer cells,^22−24^ turning tumor BL “on”. In fact, this in situ luciferin formation approach has been successfully applied for dual-analyte imaging in inflammation and tumor mice models.^25,26^ And in this study, we found it was also well-performed in precise cancer autophagy BL imaging.

## Results and Discussion

**Figure 1:**
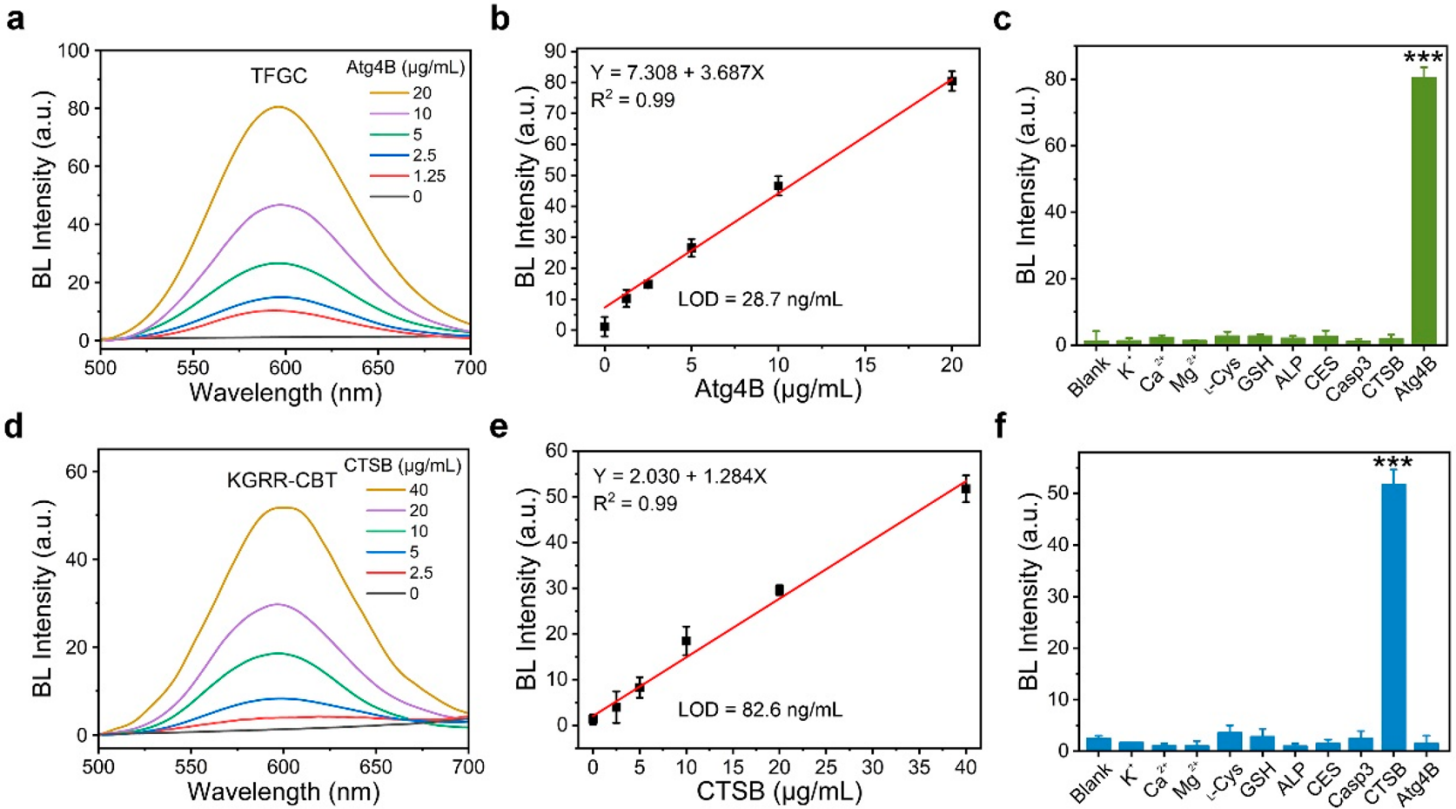
(a) BL spectra of 25 μM TFGC after incubation with different concentrations of Atg4B (0–20 μg/mL) in the presence of CBT, ATP, Mg^2+^, and fLuc. (b) The fitted calibration curve of TFGC BL intensity at 590 nm with Atg4B concentration in the region of 0–20 μg/mL. (c) BL intensity of TFGC (25 μM) after incubation with various substances: K^+^ (1 mM), Ca^2+^ (1 mM), Mg^2+^ (1 mM), l-Cys (50 μM), GSH (1 mM), ALP (100 U/L), Casp3 (30 U/L), CTSB (40 μg/mL), or Atg4B (20 μg/mL) in the presence of CBT, fLuc, and ATP. Emission: 590 nm. (d) BL spectra of 25 μM KGRR-CBT in the presence of d-Cys, fLuc, and ATP after incubation with different concentrations of CTSB (0–40 μg/mL). (e) The fitted calibration curve of KGRR-CBT BL intensity at 590 nm with CTSB concentration in the region of 0–40 μg/mL. (f) BL intensity of KGRR-CBT (25 μM) in the presence of d-Cys, fLuc, and ATP after incubation with various substances: K^+^ (1 mM), Ca^2+^ (1 mM), Mg^2+^ (1 mM), l-Cys (50 μM), GSH (1 mM), ALP (100 U/L), Casp3 (30 U/L), CTSB (40 μg/mL), or Atg4B (20 μg/mL). Emission: 590 nm.

We first synthesized and characterized the two compounds (i.e., TFGC and KGRR-CBT) and the Atg4B-cleaved product TFG (Schemes S1–S4 and Figures S1–S15). Then, we testified the responsiveness and selectivity of these two probes toward Atg4B and CTSB, respectively. High-performance liquid chromatography (HPLC) and electrospray ionization mass spectrometry (ESI-MS) analyses revealed that about 70.3% of TFGC (25 μM) was converted to TFG after incubation with 20 μg/mL of Atg4B in Tris-HCl buffer at 37 °C and pH 8.0 for 4 h (Figures S16 and S17), suggesting an efficient cleavage of TFGC by Atg4B under physiological conditions. We then validated whether the Atg4B-treated TFGC could react with CBT to turn BL “on”. After incubating 25 μM TFGC with different concentrations of Atg4B (0–20 μg/mL) in Tris-HCl buffer at 37 °C for 4 h, 50 μM CBT was added and incubated for another 0.5 h, followed by the addition of 1 mM adenosine triphosphate (ATP), 10 mM Mg^2+^, and 0.1 mg/mL firefly luciferase (fLuc), and BL spectra of the incubation solution were recorded. As expected, with the increase of Atg4B concentration, increased BL emissions centered at 590 nm were observed (Figure 1a). These results suggested that Atg4B could activate TFGC to react with CBT to yield d-aminoluciferin for BL generation. It was also observed that the BL intensities at 590 nm exhibited a linear relationship (*Y* = 7.308 + 3.687*X*, *R*^2^ = 0.99) within the Atg4B concentrations ranging from 0 to 20 μg/mL (Figure 1b). With the 3σ method (3σ/*s*),^27^ the limit of detection (LOD) of TFGC toward Atg4B was calculated to be 28.7 ng/mL (Table S1). We then investigated the kinetic parameters (i.e., turnover number *k*_cat_, Michaelis constant *K*_m_) of TFGC toward Atg4B according to the reference.^28^ The *k*_cat_/*K*_m_ value of TFGC was calculated to be 0.032 μM^–1^ s^–1^, which was comparable to those of other reported Atg4B-catalyzed reactions (Figure S18, Table S3). Afterward, we tested the selectivity of TFGC toward Atg4B against other common substances in the model cell line MDA-MB-231 in this study, including cations (e.g., K^+^, Ca^2+^, Mg^2+^), biomolecules (e.g., l-cysteine (l-Cys), glutathione (GSH)), and enzymes (e.g., alkaline phosphatase (ALP), carboxylesterase (CES), caspase 3 (Casp 3), and CTSB). Atg4B and these substances were parallelly incubated with 25 μM TFGC in Tris-HCl buffer at 37 °C for 4 h, followed by monitoring the BL intensities at 590 nm using the above-mentioned method. As shown in Figure 1c, only the Atg4B-treated group exhibited a significant BL signal, demonstrating that Atg4B could activate TFGC to react with CBT to turn BL “on” with high selectivity.

With a similar method, we then validated the responsiveness and selectivity of KGRR-CBT toward CTSB. HPLC and ESI-MS analysis revealed about 26.3% KGRR-CBT (25 μM) was converted to CBT after treatment with 40 μg/mL CTSB at 37 °C for 4 h (Figures S19 and S20), suggesting an effective KGRR-CBT-to-CBT conversion by CTSB. To validate its BL capacity, 25 μM KGRR-CBT was first treated with CTSB at concentrations ranging from 0 to 40 μg/mL at 37 °C for 4 h, then incubated with 50 μM d-Cys for 0.5 h, followed by the addition of ATP, Mg^2+^, and fLuc; finally, their BL spectra were recorded. It was observed that CTSB treatment led to concentration-dependent BL generation of KGRR-CBT (Figure 1d). The BL intensity at 590 nm and CTSB concentration within 40 μg/mL exhibited a linear relationship (*Y* = 2.030 + 1.284*X*, *R*^2^ = 0.99; Figure 1e). The LOD of KGRR-CBT toward CTSB was calculated to be 82.6 ng/mL, which is comparable to those of reported probes (Tables S2). The *k*_cat_/*K*_m_ value of KGRR-CBT was calculated to be 0.020 μM^–1^ s^–1^ (Figure S21, Table S4). Moreover, KGRR-CBT also showed high selectivity toward CTSB among the relative substances tested (Figure 1f). Additionally, the activation of KGRR-CBT by CTSB was not affected by the condensation reaction between KGRR-CBT and d-Cys (Figure S22). Collectively, these results demonstrated that, in the presence of d-Cys, CTSB could activate KGRR-CBT to illuminate BL with high responsiveness, sensitivity, and selectivity.

**Figure 2:**
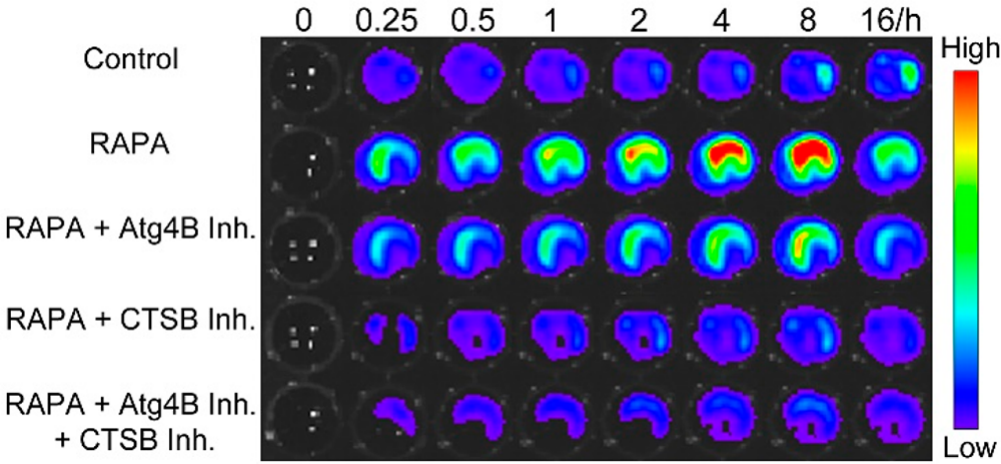
Time-course BL images of fLuc-transfected MDA-MB-231 cells with (or without) RAPA treatment incubated with TFGC (25 μM) and KGRR-CBT (25 μM). The images were acquired at 0, 0.25, 0.5, 1, 2, 4, 8, and 16 h.

Before applying TFGC and KGRR-CBT to visualize autophagy in living cancer cells, we first validated their AND-type property for Atg4B and CTSB detection in vitro. We simultaneously incubated TFGC and KGRR-CBT with 20 μg/mL Atg4B and 40 μg/mL CTSB at 37 °C for 4 h. In this case, a strong BL signal was observed (Figure S23). In contrast, much weaker BL signals were observed from the mixture of TFGC and KGRR-CBT alone, or in the presence of either Atg4B or CTSB (Figure S23). These results demonstrated that TFGC and KGRR-CBT could act as an AND-type molecular logic system for precise BL imaging of autophagy. Afterward, an autophagy-activated cancer cell model was first established by pretreating the breast cancer MDA-MB-231 cells with a widely used autophagy inducer rapamycin (RAPA).^29^ RAPA treatment (10 μM, 4 h) led to the increased levels of Atg4B and CTSB in MDA-MB-231 cells which were 2.71-fold and 1.92-fold those in untreated cells, respectively (Figure S24a and b). Moreover, after RAPA treatment, the number of LC3 fluorescent spots was increased (Figure S24c), and the characteristic autolysosomes were observed inside the cells (Figure S24d), suggesting autophagy of MDA-MB-231 cells was activated upon RAPA treatment. After confirming the stability of TFGC and KGRR-CBT under physiological conditions (Figure S25), we then testified the BL generation capacity of TFGC and KGRR-CBT in the autophagy cell model. TFGC and KGRR-CBT at 25 μM, which showed negligible cytotoxicity (Figures S26 and S27), were added to the fLuc-transfected MDA-MB-231 cells with (or without) RAPA treatment. Cells without RAPA treatment were designated as the “control” group. The RAPA-pretreated cells were further divided into four groups: (1) cells incubated with TFGC and KGRR-CBT as “RAPA”; (2) cells pretreated with an Atg4B inhibitor NCS 185058 at 1 mM for 30 min, followed by incubation with TFGC and KGRR-CBT as “RAPA + Atg4B Inh”; (3) cells pretreated with a CTSB inhibitor CA-074-Me at 2 mM for 30 min, followed by incubation of TFGC and KGRR-CBT as “RAPA + CTSB Inh”; (4) cells pretreated with both inhibitors, followed by incubation with TFGC and KGRR-CBT as “RAPA + Atg4B Inh + CTSB Inh”. As shown in Figure 2, the BL images showed that, without RAPA treatment to induce autophagy (i.e., “control” group), cells treated with TFGC and KGRR-CBT only exhibited negligible BL signals, suggesting very weak activities of Atg4B and CTSB in normal cells. In contrast, RAPA-pretreated cells (i.e., “RAPA” group) exhibited increased BL emissions with time with a maximum intensity at 8 h, indicating efficient cleavages of TFGC and KGRR-CBT by the activated Atg4B and CTSB in autophagy cells. Parallelly, with the introduction of either or both enzyme inhibitors (i.e., “RAPA + Atg4B Inh” group, “RAPA + CTSB Inh” group, and “RAPA + Atg4B Inh + CTSB Inh” group), BL emissions from the cells were largely or almost completely suppressed, suggesting the necessity of both the autophagy enzymes Atg4B and CTSB to cleave their corresponding substrates TFGC and KGRR-CBT, respectively. Notably, BL signals were also observed in “RAPA + Atg4B Inh” group. This was probably because, despite Atg4B being inhibited to cleave TFGC to generate d-Cys, endogeneous l-Cys also reacted with the CBT (released from KGRR-CBT after CTSB cleavage) to yield nonbioluminescent l-aminoluciferin,^30^ which was converted to bioluminescent d-aminoluciferin by some coenzymes (e.g., coenzyme A) in living cells (Figure S28).^25,31−33^ The time-course BL intensities in the above groups were further derived. As shown in Figure S29, for most of the groups, BL intensities increased with time and reached the plateau at 8 h, followed by attenuation at 16 h. Notably, at 8 h, the group “RAPA” showed the highest BL intensity, which was 3.74, 1.66, 4.17, or 4.29-fold that of the “control”, “RAPA + Atg4B Inh”, “RAPA + CTSB Inh”, or “RAPA + Atg4B Inh + CTSB Inh” group, respectively. Taken together, TFGC and KGRR-CBT could be efficiently cleaved by the activated enzymes Atg4B and CTSB in autophagy MDA-MB-231 cells.

**Figure 3:**
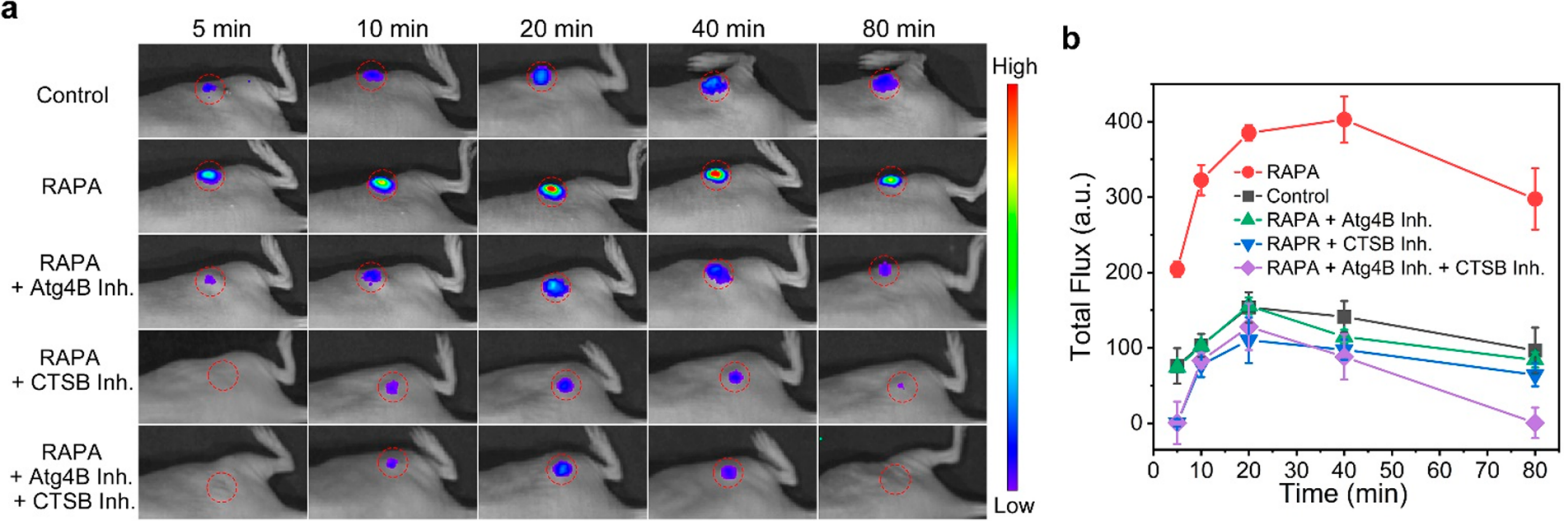
(a) Time-course BL images of mice bearing fLuc-transfected MDA-MB-231 tumors after different pretreatments and then i.p. injected with 12.5 μmol/kg TFGC and 12.5 μmol/kg KGRR-CBT. (b) Time-course quantified total flux of the images in a.

Encouraged by the above results, we finally applied TFGC and KGRR-CBT for BL imaging of autophagy in vivo. A tumor-xenografted mouse model was established through subcutaneous injection of fLuc-transfected MDA-MB-231 cells in the flank of BALB/c nude mice. After 10 days, the mice were randomly divided into five groups: (1) the “control” group, whose mice were intraperitoneally (i.p.) injected with 12.5 μmol/kg TFGC and 12.5 μmol/kg KGRR-CBT but without RAPA pretreatment; (2) the “RAPA” group, whose mice were intratumorally (i.t.) preinjected with 5 μmol/kg RAPA for 4 h, followed by i.p. injection of 12.5 μmol/kg TFGC and 12.5 μmol/kg KGRR-CBT; (3) the “RAPA + Atg4B Inh” group, whose mice were i.t. injected with 0.5 mmol/kg CA-074-Me for 0.5 h after RAPA treatment, followed by i.p. injection of 12.5 μmol/kg TFGC and 12.5 μmol/kg KGRR-CBT; (4) the “RAPA + CTSB Inh” group, whose mice were i.t. injected with 0.25 mmol/kg NCS 185058 for 0.5 h after RAPA treatment, followed by i.p. injection of 12.5 μmol/kg TFGC and 12.5 μmol/kg KGRR-CBT; (5) the “RAPA + Atg4B Inh + CTSB Inh” group, whose mice were i.t. injected with both enzyme inhibitors for 0.5 h after RAPA treatment, followed by i.p. injection of 12.5 μmol/kg TFGC and 12.5 μmol/kg KGRR-CBT. The time-course BL profiles of each mouse were then recorded with a small animal imaging system. As shown in Figure 3a and Figure S30, in mice of the “control” group, the tumor region exhibited low-level BL signals with negligible changes during 80 min of observation. The weak BL signal might be attributed to the deep cancer cells that were undergoing autophagy for survival under hypoxia and metabolic stress conditions.^34^ In contrast, the tumor region in the “RAPA” group exhibited significantly brighter BL signals, which increased with time, reached the plateau at 40 min, and then attenuated. This suggested successful activation of TFGC and KGRR-CBT by the activated Atg4B and CTSB in autophagy tumors. In contrast, the tumor regions in the other three inhibitor-treated groups (i.e., “RAPA + Atg4B Inh”, “RAPA + CTSB Inh”, and “RAPA + Atg4B Inh + CTSB Inh” groups), showed similar imaging trends but much weaker BL intensities. Notably, tumors in the “RAPA”group showed the highest BL intensity at 40 min, which was 2.85, 3.51, 4.13, or 4.57-fold greater than that of “control”, “RAPA + Atg4B Inh”, “RAPA + CTSB Inh”, and “RAPA + Atg4B Inh + CTSB Inh” groups, respectively (Figure 3b). These results collectively validated the successful application of TFGC and KGRR-CBT for dual-enzyme-activated BL imaging of autophagy in vivo with improved precision.

## Conclusion

In summary, we successfully developed two compounds, TFGC and KGRR-CBT, that responded to dual autophagy enzymes (i.e., Atg4B and CTSB) for precise autophagy BL imaging in vitro and in vivo. Upon the enzymatic cleavages by Atg4B and CTSB, TFGC and KGRR-CBT were converted to luciferin precursors d-Cys and CBT, respectively. Only in the presence of the two precursors and the two autophagy enzymes, was d-aminoluciferin generated in situ, turning BL “on”. This dual enzyme-triggered AND-type luciferin formation strategy enabled sensitive and selective imaging of these two autophagy enzymes in vitro and significantly improved the precision of autophagy imaging in cells and in vivo. And we anticipate TFGC and KGRR-CBT being applied for precise diagnosis of autophagy-related diseases in the near future.

## Supporting Information Available

Experimental procedures; detailed syntheses and characterization of KGRR-CBT, TFGC, and TFG; Schemes S1–S4; Figures S1–S30; Tables S1–S4 (PDF)
