# Supplementary material for: Atg4B and Cathepsin B-Triggered in Situ Luciferin Formation for Precise Cancer Autophagy Bioluminescence Imaging
Source: ACS Cent Sci. 2023 Nov 8;9(12):2251–6. doi: 10.1021/acscentsci.3c00696 (PMC10755845; doi:10.1021/acscentsci.3c00696)
Supplement: Supplementary file 1 — oc3c00696_si_001.pdf [file oc3c00696_si_001.pdf]

Supporting Information

Atg4B and Cathepsin B-Triggered in Situ Luciferin Formation for Precise Cancer Autophagy Bioluminescence Imaging

Xiaotong Cheng,† Tiantian Xia,† Xianbao Sun, Guowei Liang, Xiaoyang Liu, and Gaolin Liang*

State Key Laboratory of Digital Medical Engineering, School of Biological Science and Medical Engineering, Southeast University, Nanjing 210096, China

†Xiaotong Cheng and Tiantian Xia contributed equally.

Correspondence and requests for materials should be addressed to e-mail: gliang@seu.edu.cn (G. Liang)

Contents:

1. Experimental procedures

2. Syntheses and characterizations

3. Supporting figures and tables

4. References

1. Experimental procedures

General methods

All the starting materials were obtained from Aladdin Chemistry Co., Ltd. (China), GL Biochem Co., Ltd. (Shanghai), Sigma-Aldrich (Shanghai, China). Commercially available reagents were used without further purification, unless noted otherwise. All other chemicals were analytical reagent grade or better. Deionized (DI) water (18.2 MΩ·cm) was purified by a Milli-Q system (Millipore) and used throughout the experiments. Atg4B was purchased from Abnova (Taiwan, China). Cathepsin B (CTSB) was obtained from Bio-Techne (USA). Dulbecco's modified Eagle's medium (DMEM) were purchased from KeyGen Biotech (Nanjing, China). Fetal bovine serum (FBS, Australia origin) was obtained from Gibco (USA). High performance liquid chromatography (HPLC) analyses were performed on an Agilent 1260 HPLC system equipped with a G1322A pump and in-line diode array ultraviolet (UV) detector using an Agilent Zorbax 300SB-C18 RP column with CH_3CN (0.1% of trifluoroacetic acid (TFA)) and water (0.1% of TFA) as the eluent. The spectra of electrospray ionization-mass spectrometry (ESI-MS) were obtained on a Finnigan LCQ Advantage ion trap mass spectrometer (ThermoFisher Corporation) that was equipped with a standard ESI source. Fluorescence imaging was conducted on a fluorescence microscope (Ti2-U, Nikon, Japan). Flow cytometry was conducted with a flow cytometer (NovoCyte 2070R, ACEA Biosciences, Inc., USA). The biological transmission electron microscopy (bio-TEM) images were characterized under the transmission electron microscope (H 600(4), Hitachi, Japan). Bioluminescence images of cells and animals were captured by a PerkinElmer animal imaging system.

Expression of firefly luciferase (fLuc)

The expression and purification protocol of fLuc was according to the literature method.[1] In detail, BL 21 cells were transformed with the appropriate plasmids (pET-6xHis/Luciferase) and cultured in lysogeny broth (LB) medium supplemented with 100 μg/mL ampicillin at 37 °C until their OD600 reached 0.6-0.8. Afterwards, the BL 21 cells were induced with 0.5 mM isopropyl-β-d-thiogalactopyranoside (IPTG) at 16 °C for 20 h. The bacterial cells were then

precipitated (4500 rpm, 10 min) and re-suspended in binding/wash buffer (Sangon Biotech, China). After disrupting the bacterial cells via sonication on ice. The His-tagged enzymes were purified using Ni-NTA-agarose (Sangon Biotech, China) equilibrated in TBS buffer (50 mM Tris, 150 mM NaCl, pH 8.0) supplemented with 10 mM imidazole. After the enzyme supernatant was loaded in the Ni-NTA column, rotated for 30 min, and the column was washed with binding/wash buffer for 3 times. Then, the enzyme was eluted out by elution buffer (Sangon Biotech, China) for 3 times. The protein collected from elution after dialysis was used without further purification and the enzyme concentration was determined by BCA protein assay kit (KeyGen Biotech, China).

Cytotoxicity studies

For the cell viability assay, fLuc-transfected MDA-MB-231 cells were seeded in 96-well plates (5×10^3 per well), and cultured with cell culture medium for 24 h. Afterwards, the cell culture medium was removed and cells were treated with different concentrations of compounds of interests. The cell viabilities were further detected by 3-(4,5-dimethyl-2-thiazolyl)-2,5-diphenyl-2-H-tetrazolium bromide (MTT) assay. In detail, 10 μL MTT solution (5 mg/mL) was added into each well and the plates were incubated in the incubator for 4 h. Afterwards, 150 μL dimethyl sulfoxide (DMSO) was used to replace the reaction solution in each well. After measuring the absorbance of each well at 570 nm with multimode microplate reader (Spectra Max iD3, BD, USA), the cell viability percentage relative to the untreated cells was calculated. The MTT assay was performed in sextuplet, and the average values of the six measurements were taken.

Relative CTSB activity measurements

1×10^5 fLuc-transfected MDA-MB-231 cells with or without rapamycin (RAPA) treatment were collected and washed with ice-cold PBS for 3 times. Afterwards, the cells were resuspended in 50 μL RIPA lysis buffer (Beyotime, China) and lysed on ice for 30 min. The CTSB activity of cell lysate was measured with Cathepsin B Activity Fluorometric Assay Kit (Genmed Scientifics, China).

Western blot

After RAPA treatment, fLuc-transfected MDA-MB-231 cells were washed with PBS followed by lysing with RIPA buffer (Beyotime, China) containing 1% Protease Inhibitor Cocktail Set III (Beyotime, China) on ice for 30 min. After centrifugation, the supernatants were collected and the total protein concentration of each sample was analyzed by BCA protein assay kit (KeyGen Biotech, China). The protein samples were then added with reduced protein loading buffer and diluted to the same concentration prior to the denaturation in boiling water bath for 15 min. Afterwards, the protein samples were loaded on a 12% sodium dodecyl sulfate-polyacrylamide gel electrophoresis (SDS-PAGE) gel which was subsequently transferred to a polyvinylidene difluoride (PVDF) membrane for 120 min. After incubation with NcmBlot blocking buffer (NCM Biotech, China) at room temperature for 10 min, the PVDF membrane was washed with Tris-buffered saline (TBS) containing 0.5% Tween-20 (Solarbio, China) (TBST) for 3 times followed by the incubation with a rabbit monoclonal antibody against Atg4B (abcam, UK) (the primary antibody) at 4 °C with shaking overnight. Next, the PVDF membrane was washed with TBST and then incubated with the corresponding horse radish peroxidase (HRP)-conjugated secondary antibody at room temperature for 60 min. Finally, the target proteins were detected after incubating the PVDF membrane with enhanced chemiluminescence (ECL) reagent (Tanon, China) and visualized via a chemiluminescence imaging system (Tanon 5200, China). GAPDH was set as the loading control.

Immunofluorescence staining

FLuc-transfected MDA-MB-231 cells were treated with cell culture medium or 10 μM RAPA for 4 h. Afterwards, the cells were immobilized by 0.1% glutaraldehyde for 10 min at room temperature. Followed by the permeabilization by 0.1% Triton-X 100 for 10 min, the cells were then incubated with 3% bovine serum albumin (BSA)solution for 2 h prior to the incubation with the LC3A/B antibody (Bioss, China) in BSA solutions at 4 ºC overnight. Next, the cells were incubated with fluorescein isothiocyanate (FITC)-conjugated goat anti-rabbit IgG antibody (KeyGen Biotech, China) at room temperature for 2 h. Before confocal fluorescence imaging, the cells were washed with PBS for 3 times to remove unbound antibodies.

Animal model

Three-week-old female BALB/c nude mice were obtained from Yangzhou University Medical Center (Yangzhou, China). All experimental procedures at the animal level were performed under the approval of the Animal Care Committee of Southeast University (No.20230410031) and in compliance with the Regulations for the Administration of Affairs Concerning Experimental Animals of China. To establish tumor-bearing mouse model, 5×10^7 fLuc-transfected MDA-MB-231 cells (suspended in 100 μL of PBS) were subcutaneously injected into the right tight of each mouse. After 10 days, tumor models were established and the mice were randomly divided into five groups ($n = 4$). Each group were tumor-directly injected with PBS or 5 μmol/kg RAPA for 4 h to construct tumor autophagy-inactivated or autophagy-activated models.

Statistical analysis

Statistical analysis was conducted by the one-way analysis of variance (ANOVA) for multiple groups. P value < 0.05 is considered statistically significant (*P < 0.05, **P < 0.01, ***P < 0.001).

2. Syntheses and characterizations

Scheme S1. Synthetic route for compound **B**.

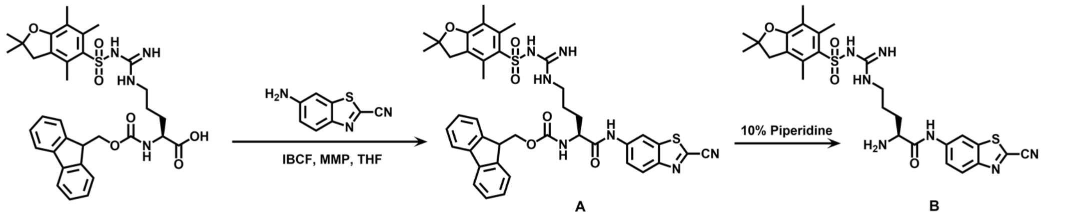

*Synthesis of compound **B***. Isobutyl chloroformate (IBCF, 66.15 μL, 0.51 mmol) was added to a mixture of Fmoc-Arg(Pbf)-OH (220.58 mg, 0.34 mmol) and 4-methylmorpholine (MMP, 74.76 μL, 0.68 mmol) in tetrahydrofuran (THF, 2 mL) at 0 °C. The reaction mixture was stirred at 0 °C for 40 min. Then, the solution of 2-cyano-6-aminobenzothiazole (CBT, 34.02 mg, 0.34 mmol) was added to the reaction mixture with further stir for 1.5 h. Then the mixture was stirred overnight at room temperature. Afterwards, compound **A** was obtained through HPLC purification using water/acetonitrile mixed solvent (volume ratio from 1:9 to 0:10) added with 0.1% TFA as the eluent (Figure S1). MS: calculated for compound **A** $[M+H]^+$: 806.3, obsvd. ESI-MS $[M+H]^+$: *m*/*z* 805.9 (Figure S2). Then, the Fmoc protecting group of compound **A** was cleaved with 10% piperidine in DMF for 10 min. Compound **B** was obtained through HPLC purification using water/acetonitrile mixed solvent (volume ratio from 6:4 to 0:10) added with 0.1% TFA as the eluent (Figure S3). MS: calculated for compound **B** $[M+H]^+$: 584.2, obsvd. ESI-MS $[M+H]^+$: *m*/*z* 584.0 (Figure S4).

Scheme S2. Synthetic route for **KGRR-CBT**.

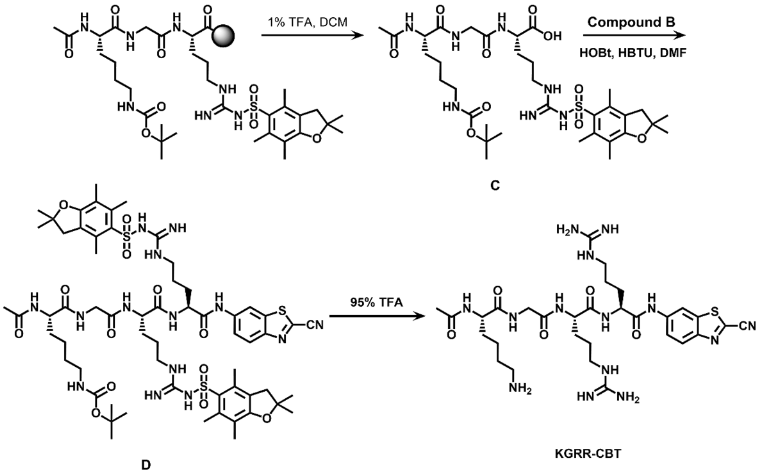

*Synthesis of **KGRR-CBT***. Solid phase peptide synthesis (SPPS, 1.0 g 2-chlorotrityl chloride resin) was used to prepare the Ac-Lys(Boc)-Gly-Arg(Pbf)-Resin. Compound **C** was obtained after cleaving the peptide from the resin and purified through HPLC using water/acetonitrile mixed solvent (volume ratio from 6:4 to 1:9) added with 0.1% TFA as the eluent. MS: calculated for compound **C** $[M+H]^+$: 754.4, obsvd. ESI-MS $[M+H]^+$: *m/z* 754.0 (Figure S5). Next, compound **B**, compound **C**, 1-hydroxybenzotrizole (HOBt), 2-(1H-benzotriazol-1-yl)-1,1,3,3,-tetramethyluronium hexafluorophosphate (HBTU), and *N, N*-diisopropylethylamine (DIPEA) were dissolved in DMF. After overnight reaction and HPLC purification, compound **D** was obtained (Figure S6). MS: calculated for **D** $[M+H]^+$: 1319.6, obsvd. ESI-MS $[M+H]^+$: *m/z* 1319.5 (Figure S7). Ac-Lys-Gly-Arg-Arg-CBT (**KGRR-CBT**)was obtained after cleaving the protecting group of compound **D** with 95% TFA. HPLC was applied to purify **KGRR-CBT** using water-acetonitrile added with 0.1% TFA as the eluent from 8:2 to 1:9 (Figure S8). MS: calculated for **KGRR-CBT** $[M+H]^+$: 715.4, obsvd. ESI-MS $[M+H]^+$: *m/z* 715.3 (Figure S9). ^{1}H NMR (500 MHz, d_6-DMSO) δ (ppm): 10.49 (d, J = 16.5 Hz, 1 H), 8.77 (dd, J = 11.9, 2.1 Hz, 1 H), 8.36–8.25 (m, 2 H), 8.23–8.12 (m, 2 H), 8.02 (dd, J = 12.8, 7.4 Hz, 1 H), 7.86 (dd, J = 7.5, 3.9 Hz, 2 H), 7.78 (d, J = 5.8 Hz, 1 H), 4.45 (dt, J = 13.3, 6.3 Hz, 1 H), 4.35 (td, J = 8.0, 4.9 Hz, 1 H), 4.16 (ddd, J = 8.9, 7.0, 5.0 Hz, 1 H), 3.77 (qd, J = 16.7, 5.5 Hz, 2 H), 3.22–3.06 (m, 4 H), 2.76 (dt, J = 11.3, 5.9 Hz, 2 H), 1.87 (s, 3 H), 1.80–1.22 (m, 14 H) (Figure S10). ^{13}C NMR (126 MHz, d_6-DMSO) δ (ppm): 172.56, 171.65, 171.07, 170.03, 169.27, 156.99 (2 C), 147.80, 139.38, 136.72, 135.12, 124.88, 120.91, 113.59, 111.42, 53.55, 53.02, 52.34, 42.20, 40.50, 40.42, 38.71, 30.98, 29.10, 28.90, 26.71, 25.34, 25.05, 22.47, 22.45 (Figure S11).

Scheme S3. Synthetic route for **TFGC**.

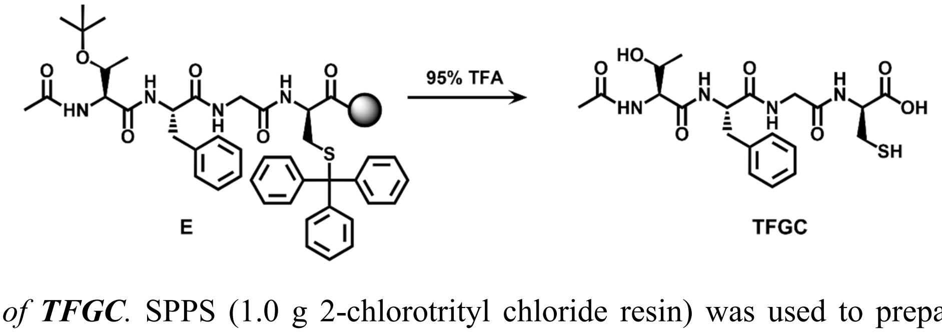

*Synthesis of **TFGC***. SPPS (1.0 g 2-chlorotrityl chloride resin) was used to prepare the Ac-Thr(OtBu)-Phe-Gly-D-Cys(Trt)-Resin (compound **E**). Ac-Thr-Phe-Gly-D-Cys (**TFGC**) was

obtained after cleaving the protecting groups and the peptide from the resin and purified through HPLC using water/acetonitrile mixed solvent (volume ratio from 7:3 to 3:7) added with 0.1% TFA as the eluent. MS: calculated for **TFGC** $[M+H]^+$: 469.2, obsvd. ESI-MS $[M+H]^+$: *m/z* 469.1 (Figure S12). ^1H NMR (300 MHz, d_6-DMSO) δ (ppm): 8.33–7.63 (m, 4 H), 7.30–7.12 (m, 5 H), 4.51 (td, J = 8.5, 4.4 Hz, 1 H), 4.40 (td, J = 7.4, 4.8 Hz, 1 H), 4.15 (dd, J = 8.2, 4.8 Hz, 2 H), 3.92–3.68 (m, 4 H), 3.05 (dd, J = 13.9, 4.6 Hz, 1 H), 2.83 (dt, J = 9.9, 3.4 Hz, 2 H), 2.42 (t, J = 8.5 Hz, 1 H), 1.85 (s, 3 H), 0.93 (d, J = 6.2 Hz, 3 H) (Figure S13). ^{13}C NMR (126 MHz, d_6-DMSO) δ (ppm): 171.36, 171.19, 170.10, 169.59, 168.67, 137.69, 129.18 (2 C), 128.04 (2 C), 126.24, 66.54, 58.22, 54.34, 53.95, 41.84, 37.27, 25.56, 22.53, 19.40 (Figure S14).

Scheme S4. Synthetic route for **TFG**.

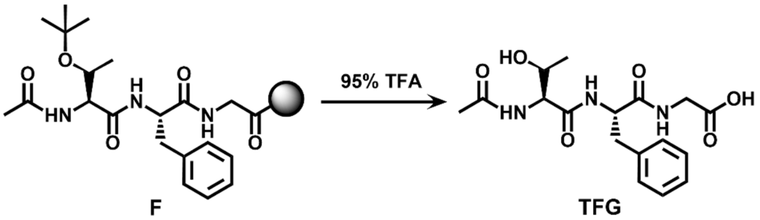

*Synthesis of **TFG***. SPPS (1.0 g 2-chlorotrityl chloride resin) was used to prepare the Ac-Thr(OtBu)-Phe-Gly-Resin (compound **F**). Ac-Thr-Phe-Gly (**TFGC**) was obtained after cleaving the protecting groups and the peptide from the resin and purified through HPLC using water/acetonitrile mixed solvent (volume ratio from 7:3 to 3:7) added with 0.1% TFA as the eluent. MS: calculated for **TFG** $[M]^+$: 365.2, obsvd. ESI-MS $[M]^+$: *m/z* 365.1 (Figure S15).

3. Supporting figures and tables

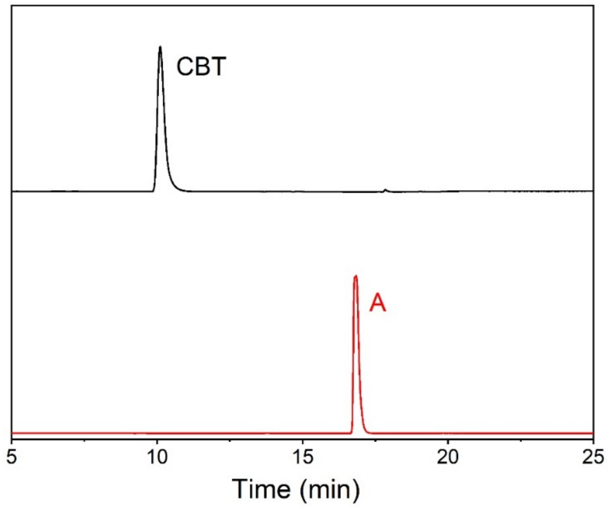

Figure S1. HPLC traces of CBT (black), and compound **A** (red).

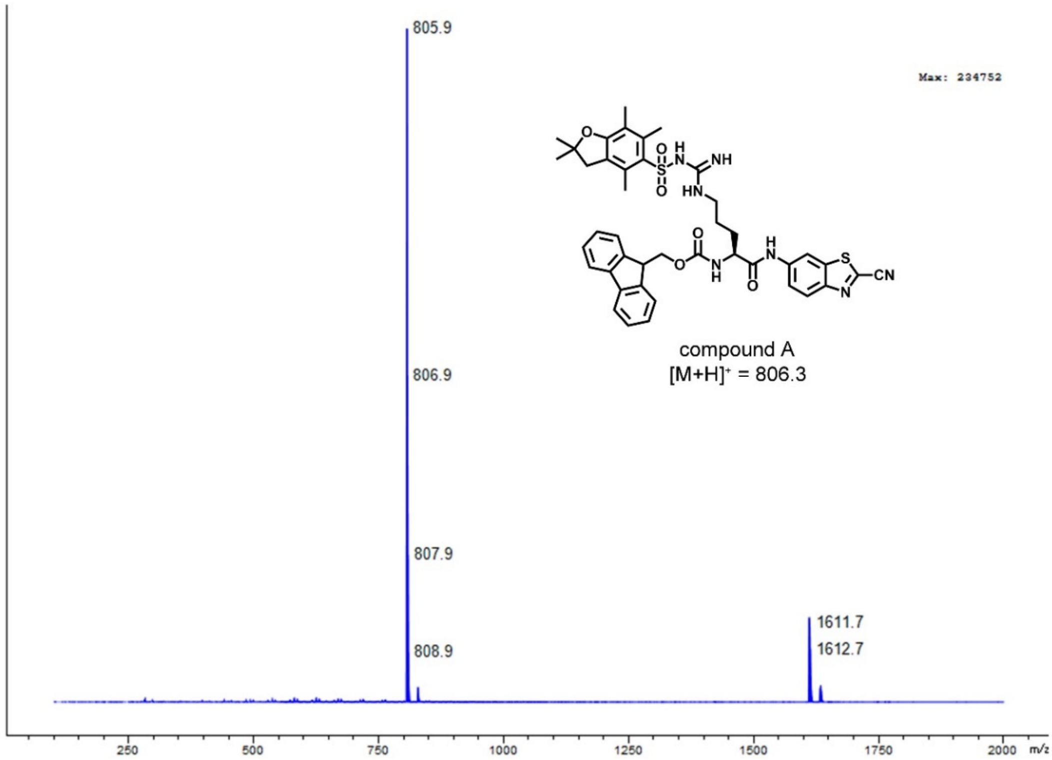

Figure S2. ESI-MS spectrum of compound **A**.

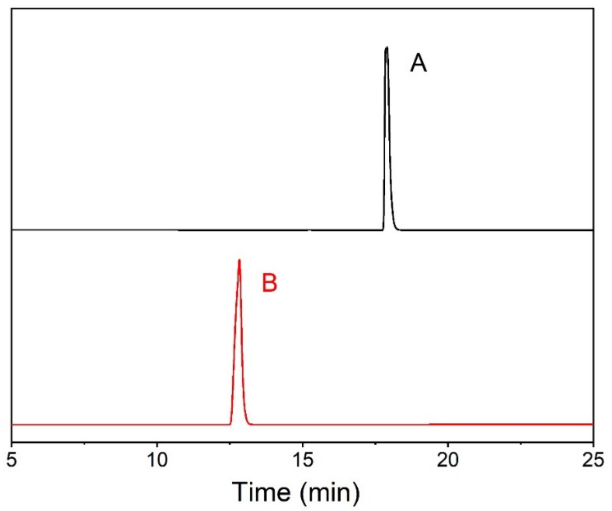

Figure S3. HPLC traces of compound **A** (black), and compound **B** (red).

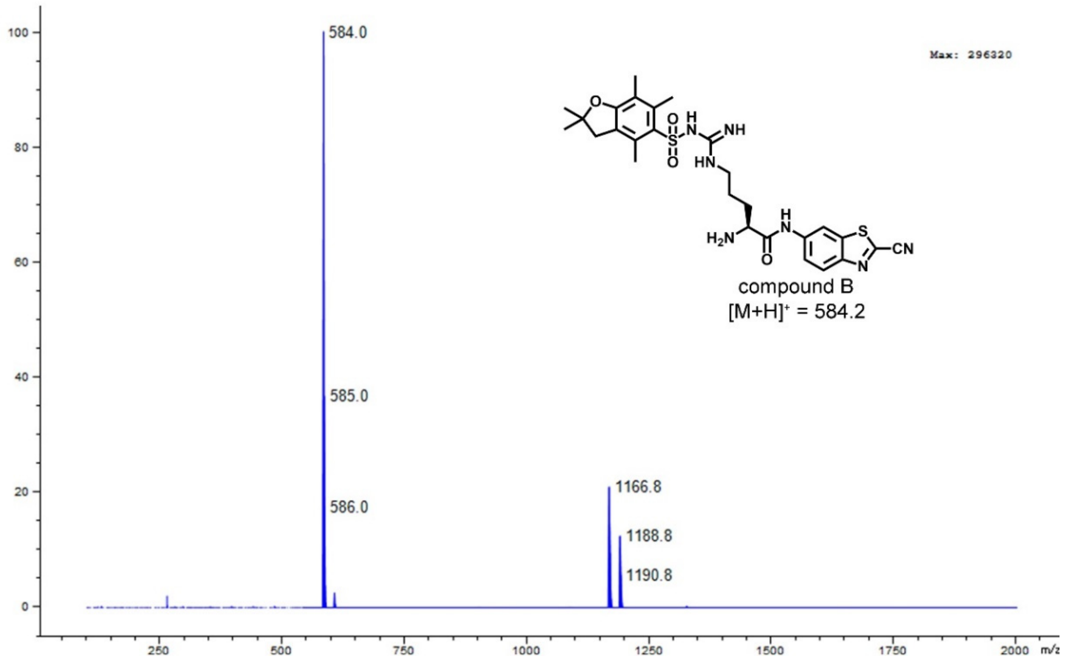

Figure S4. ESI-MS spectrum of compound **B**.

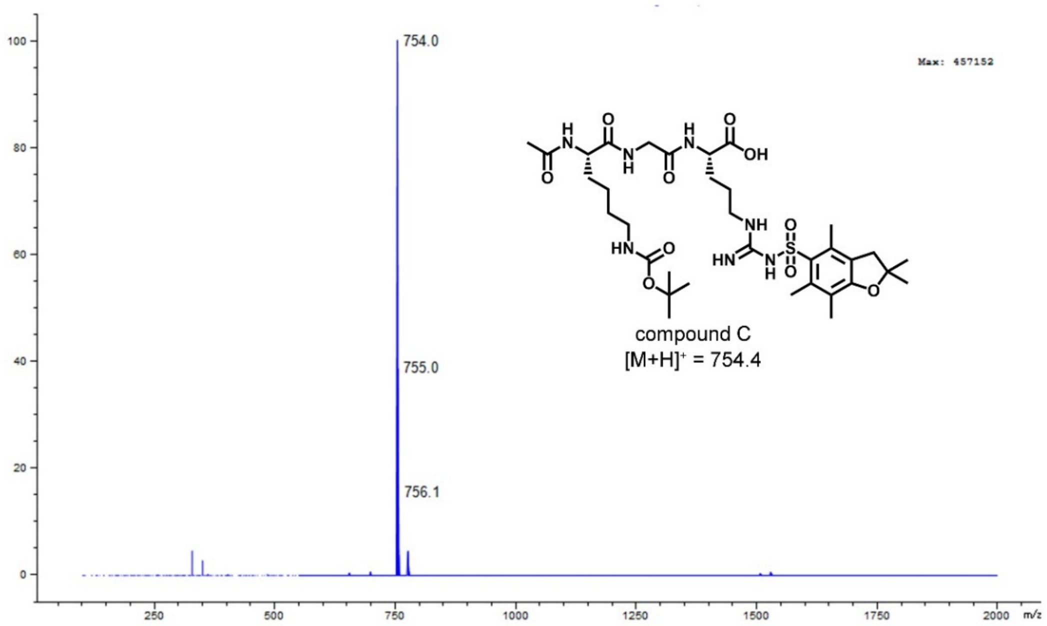

Figure S5. ESI-MS spectrum of compound **C**.

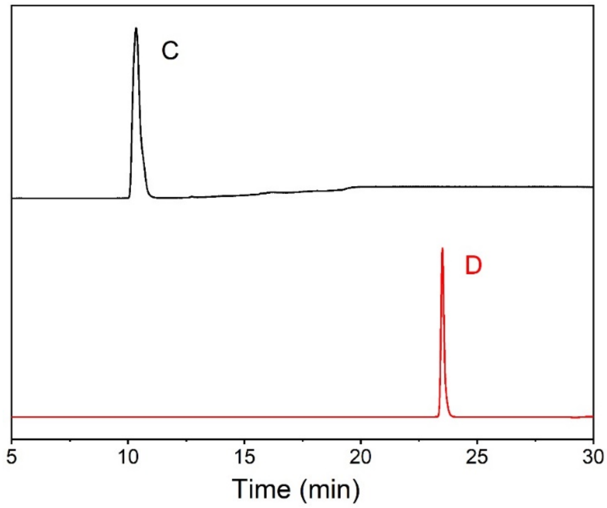

Figure S6. HPLC traces of compound **C** (black), and compound **D** (red).

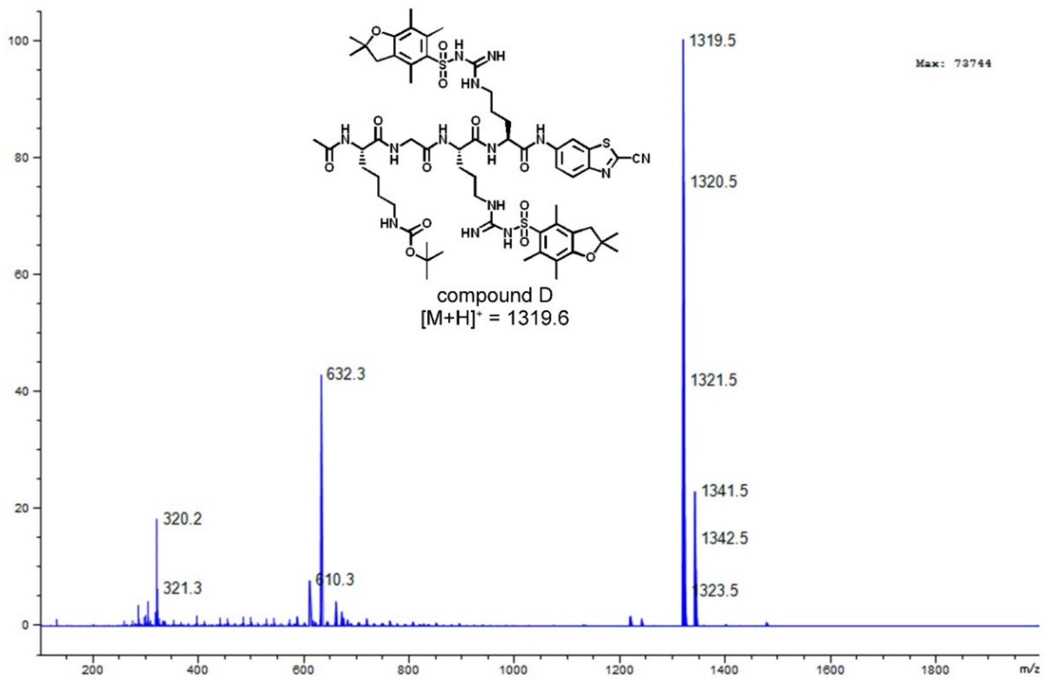

Figure S7. ESI-MS spectrum of compound **D**.

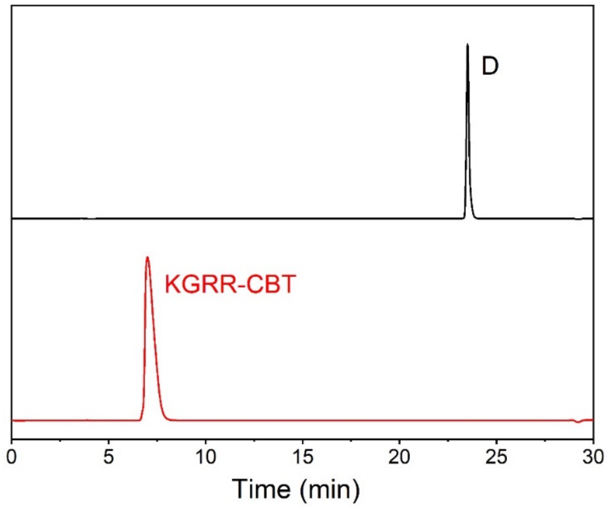

Figure S8. HPLC traces of compound **D** (black), and **KGRR-CBT** (red).

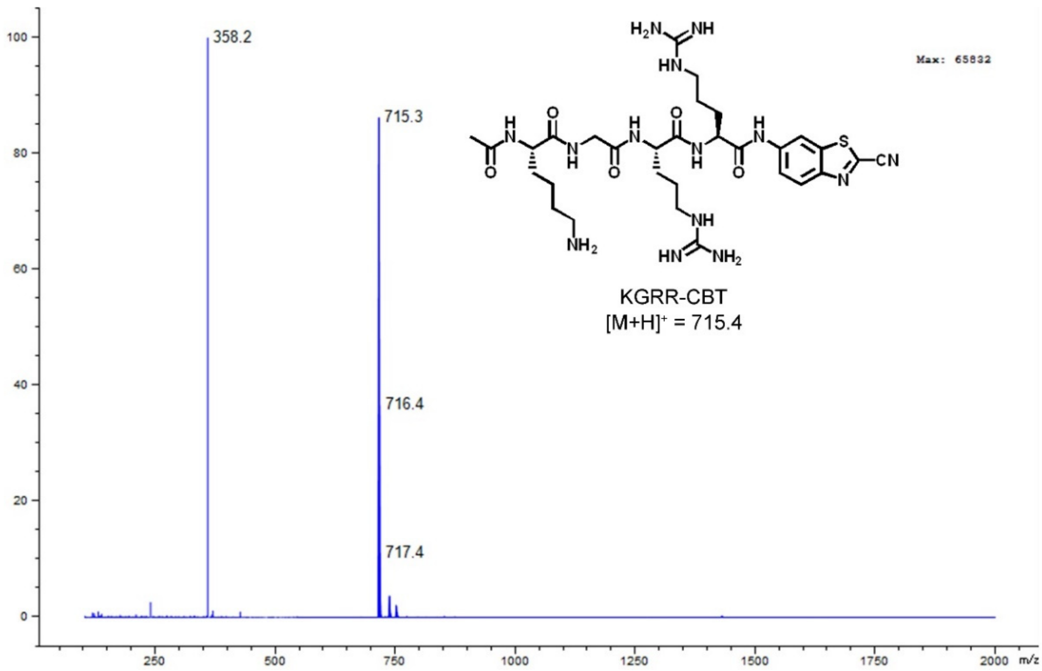

Figure S9. ESI-MS spectrum of **KGRR-CBT**.

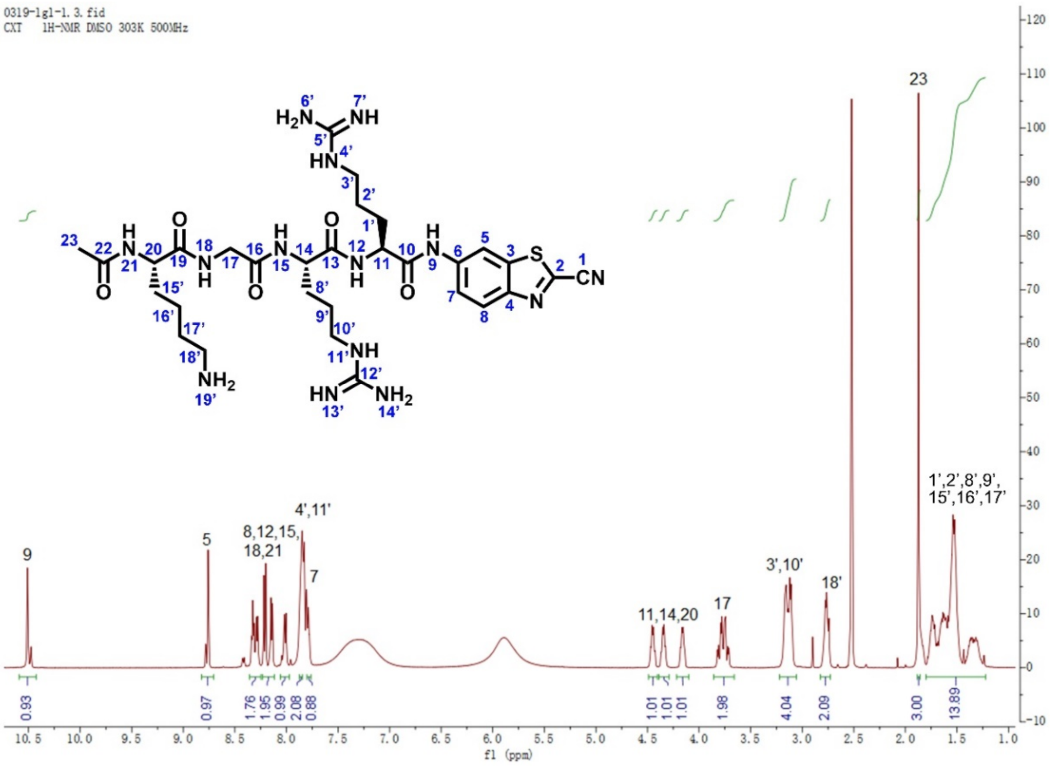

Figure S10. ^{1}H NMR spectrum of **KGRR-CBT** in d_6-DMSO.

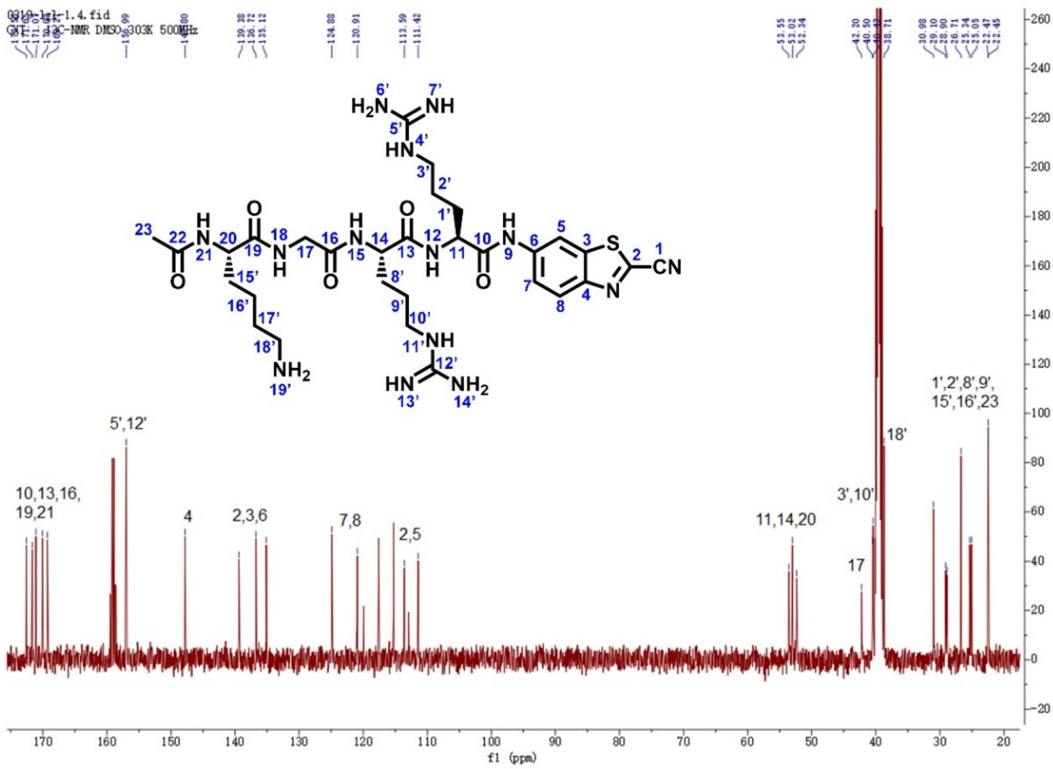

Figure S11. ^{13}C NMR spectrum of **KGRR-CBT** in d_6-DMSO.

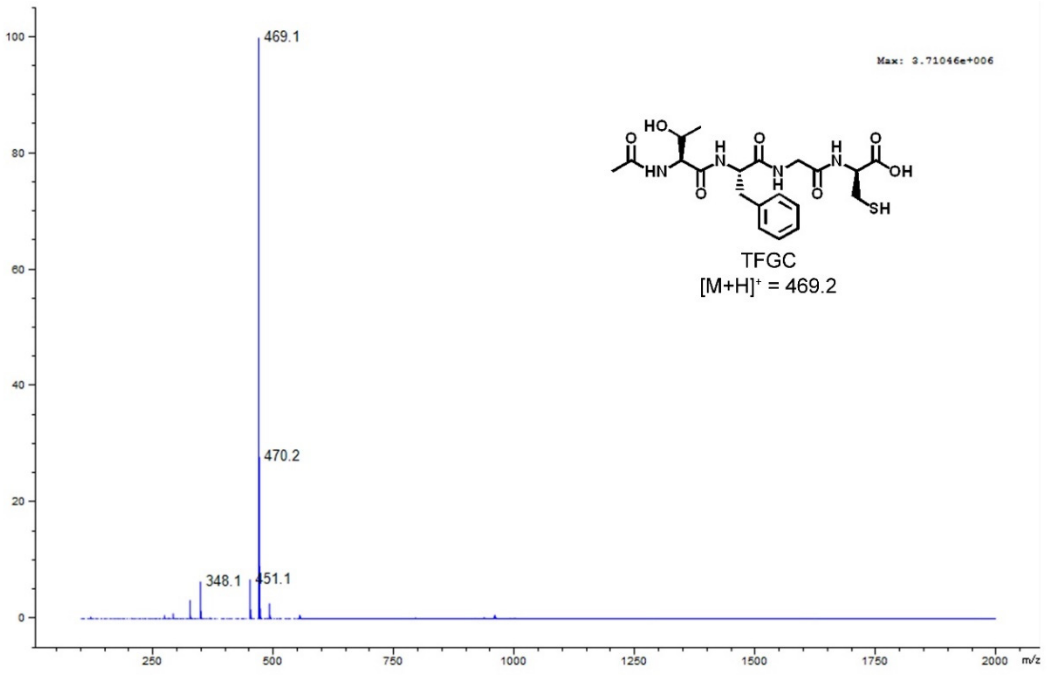

Figure S12. ESI-MS spectrum of **TFGC**.

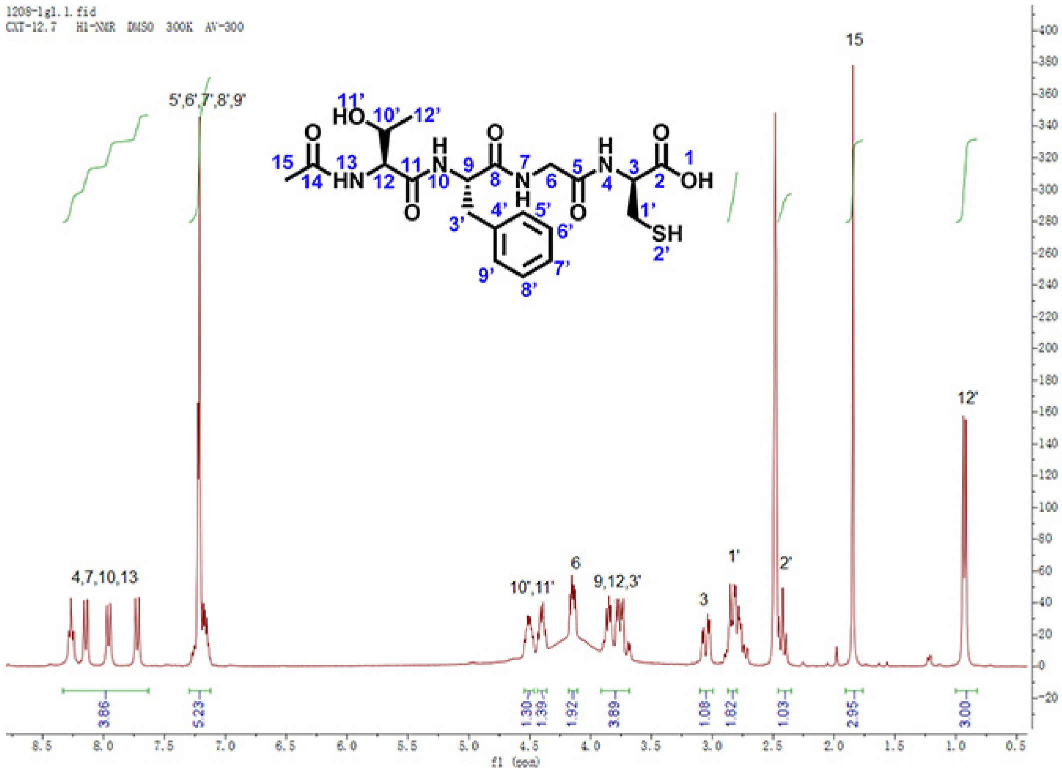

Figure S13. ^{1}H NMR spectrum of **TFGC** in d_6-DMSO.

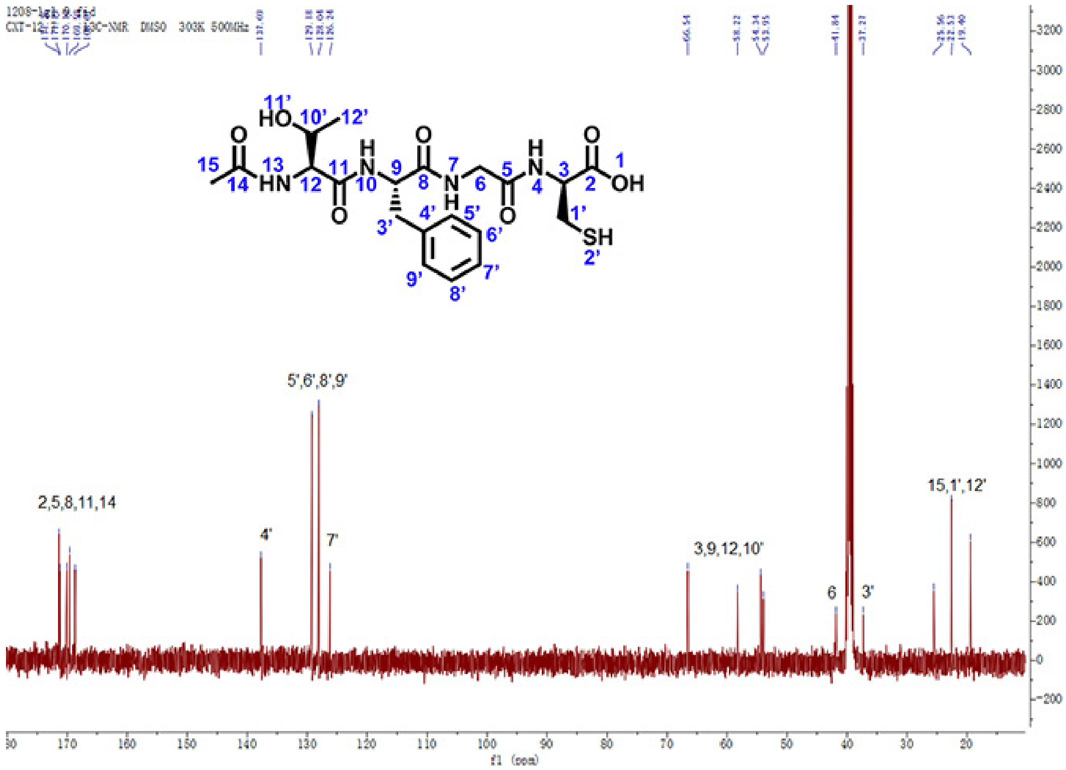

Figure S14. ^{13}C NMR spectrum of **TFGC** in d_6-DMSO.

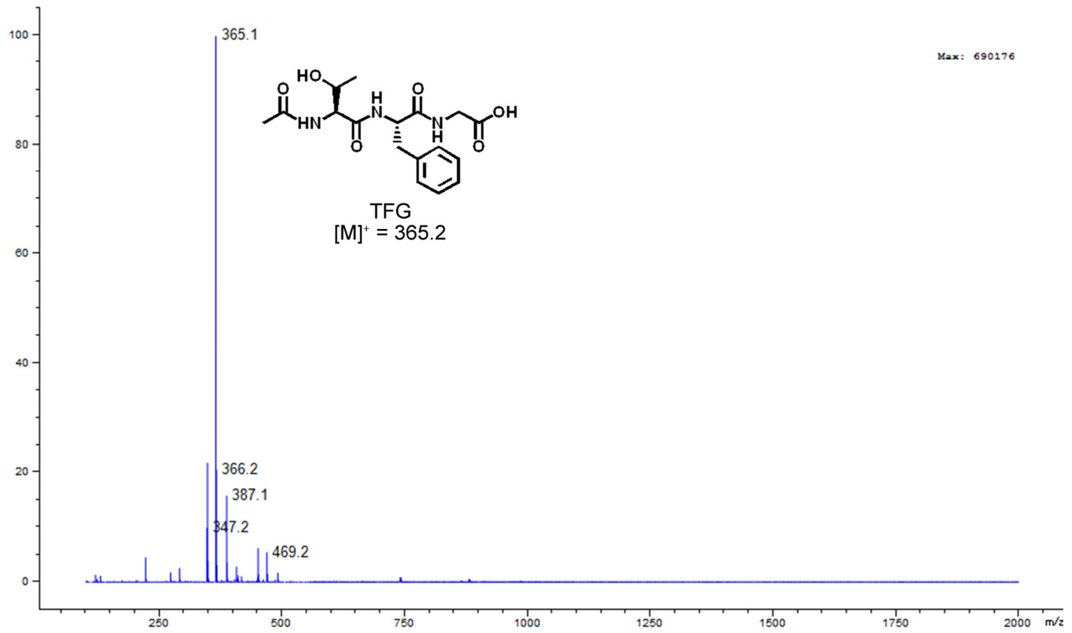

Figure S15. ESI-MS spectrum of **TFG**.

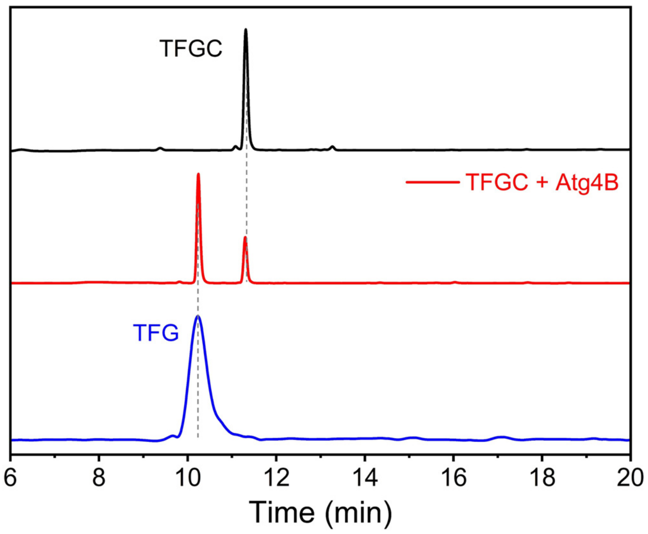

Figure S16. HPLC traces of **TFGC**, **TFG**, and **TFGC** incubated with 20 μg/mL Atg4B for 4 h at 37 °C. Detection wavelength: 254 nm.

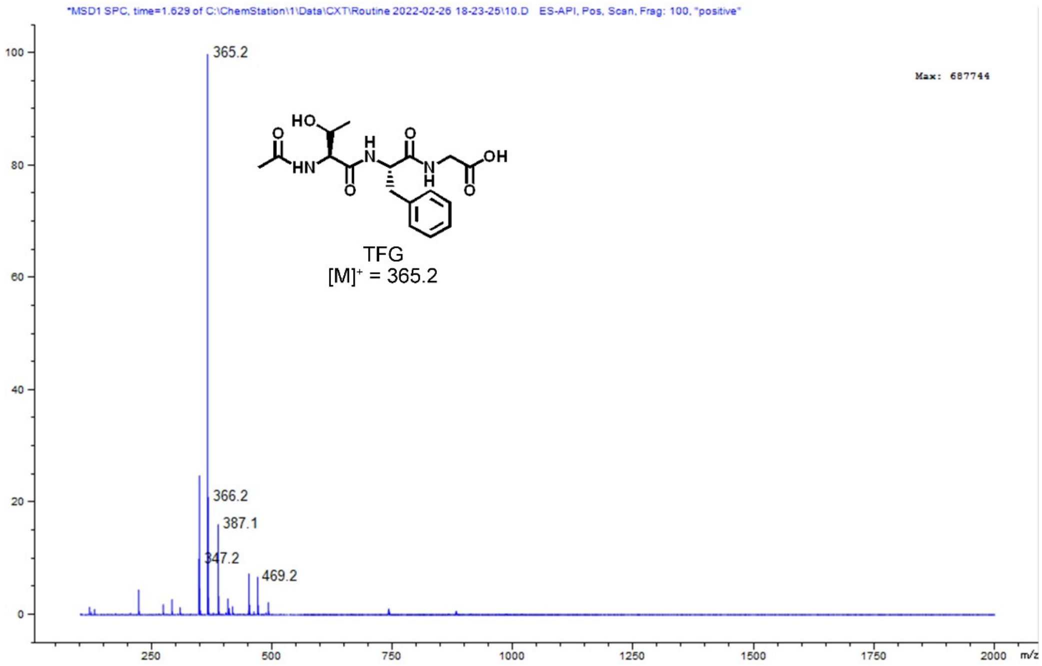

Figure S17. ESI-MS spectrum of the HPLC peak at 10.2 min in Figure S16.

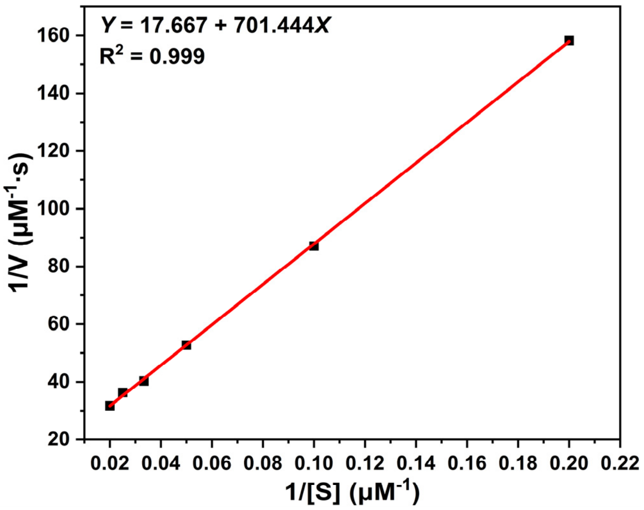

Figure S18. Lineweaver-Burk plots for the Atg4B-catalyzed reaction of **TFGC**. Conditions: 2 μg/mL Atg4B, 0−50 μM of **TFGC**.

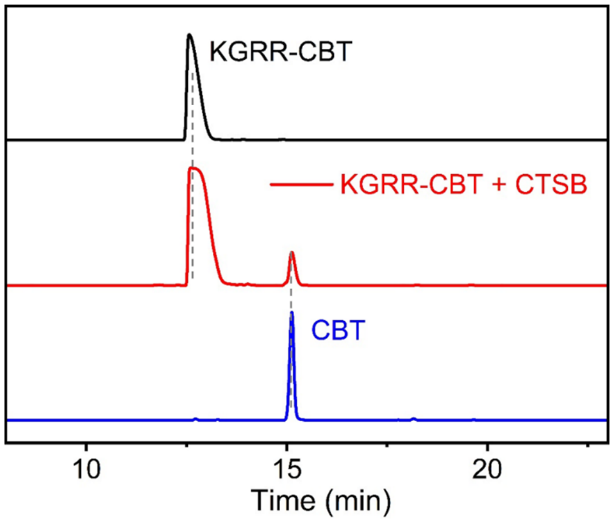

Figure S19. HPLC traces of **KGRR-CBT**, CBT, and **KGRR-CBT** incubated with 40 μg/mL CTSB for 4 h at 37 ºC. Detection wavelength: 320 nm.

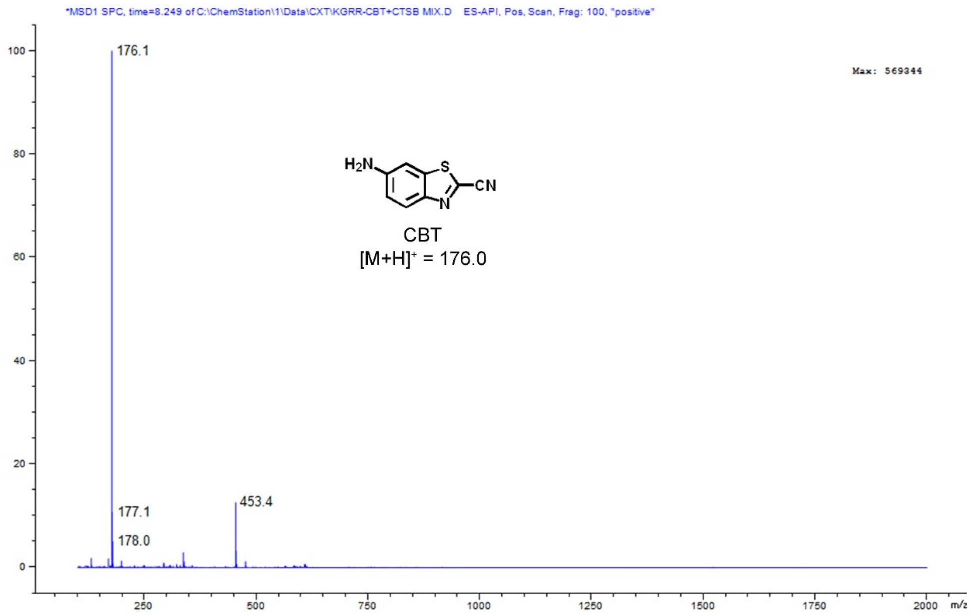

Figure S20. ESI-MS spectrum of the HPLC peak at 15.1 min in Figure S19.

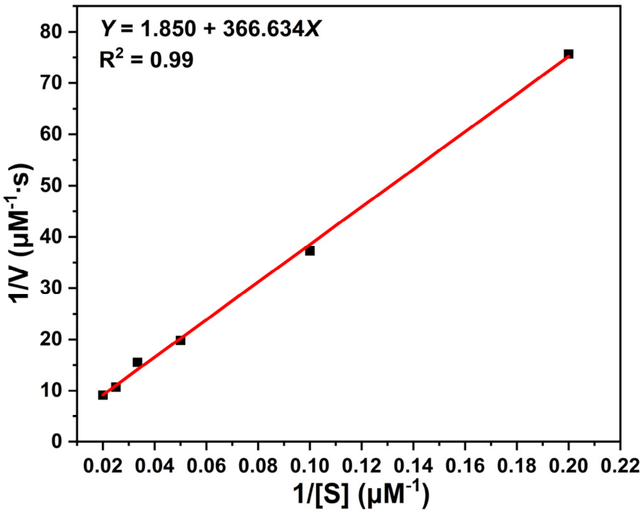

Figure S21. Lineweaver-Burk plots for the CTSB-catalyzed reaction of **KGRR-CBT**. Conditions: 5 μg/mL CTSB, 0−50 μM of **KGRR-CBT**.

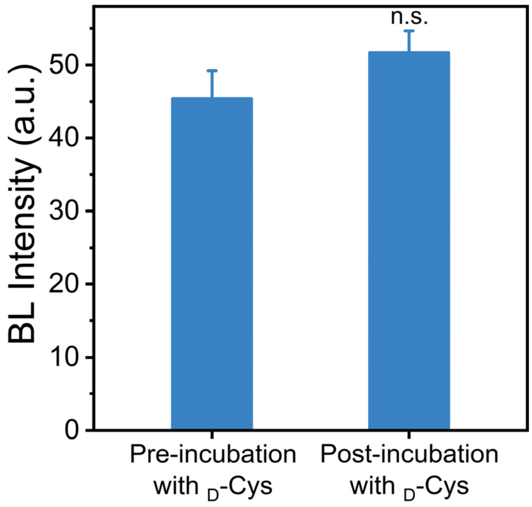

Figure S22. BL intensity of 25 μM **KGRR-CBT** pre-incubated with 50 μM D-Cys before incubation with 40 μg/mL CTSB for 4 h (left column) or post-incubated with D-Cys after incubation with 40 μg/mL CTSB (right column). The experiments were conducted in the presence of fLuc and ATP. (n.s. = no significance).

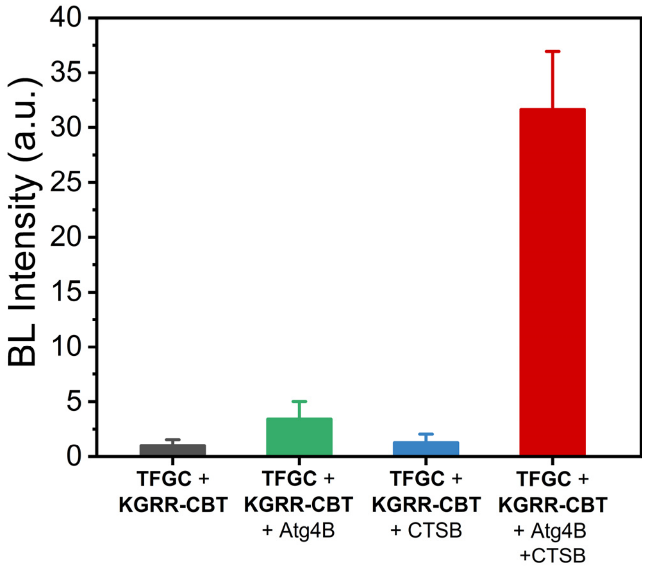

Figure S23. AND-type property of **TFGC** and **KGRR-CBT** for Atg4B and CTSB detection. To quantify aminoluciferin formation, 1 mM ATP, 10 mM Mg^{2+}, and 0.1 mg/mL fLuc were added to the **TFGC**/**KGRR-CBT** solutions.

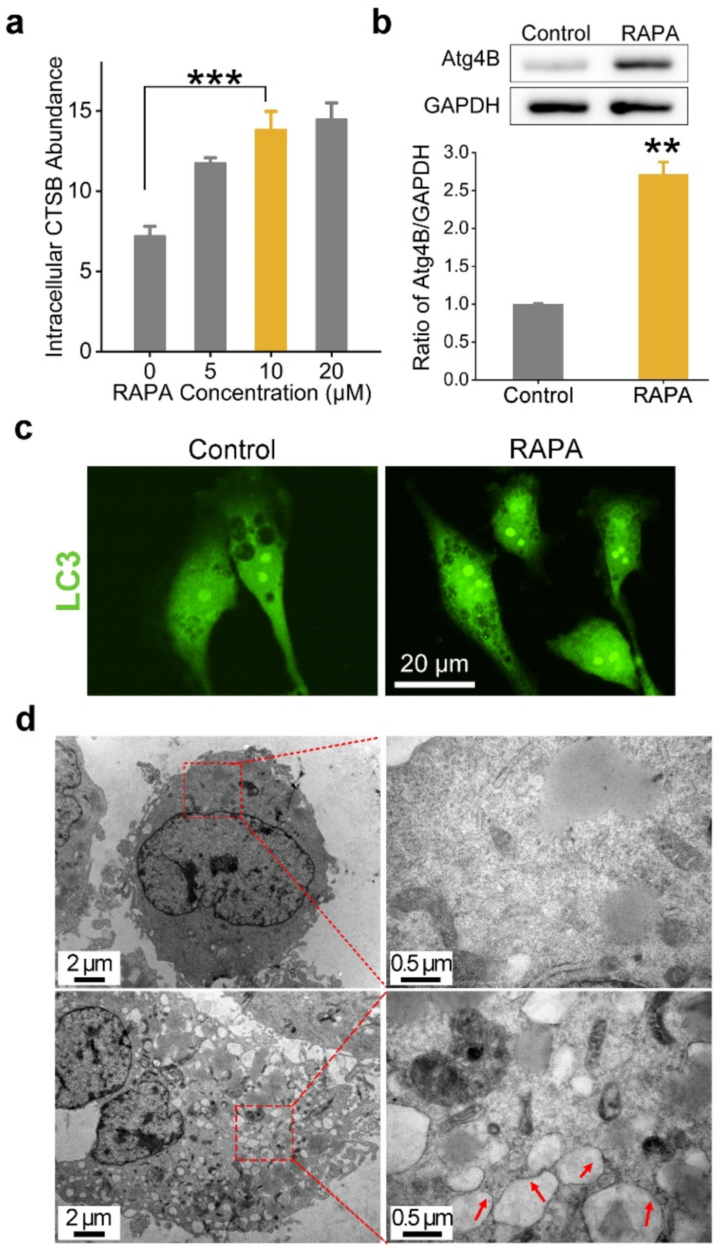

Figure S24. (a) CTSB activities of fLuc-transfected MDA-MB-231 after RAPA treatment for 4 h. (b) Western blotting of Atg4B in fLuc-transfected MDA-MB-231 cells after 10 μM RAPA treatment for 4 h and corresponding quantitative analysis of band intensity of Atg4B/GAPDH. (c) Immunofluorescence staining of fLuc-transfected MDA-MB-231 cells with (or w/o) RAPA treatment using LC3A/B antibody and FITC-labeled goat anti-rabbit IgG antibody. (d) TEM images of fLuc-transfected MDA-MB-231 cells without (the top row) or with (the bottom row) 10 μM RAPA treatment for 4 h. Red arrows indicate the locations of autolysosomes.

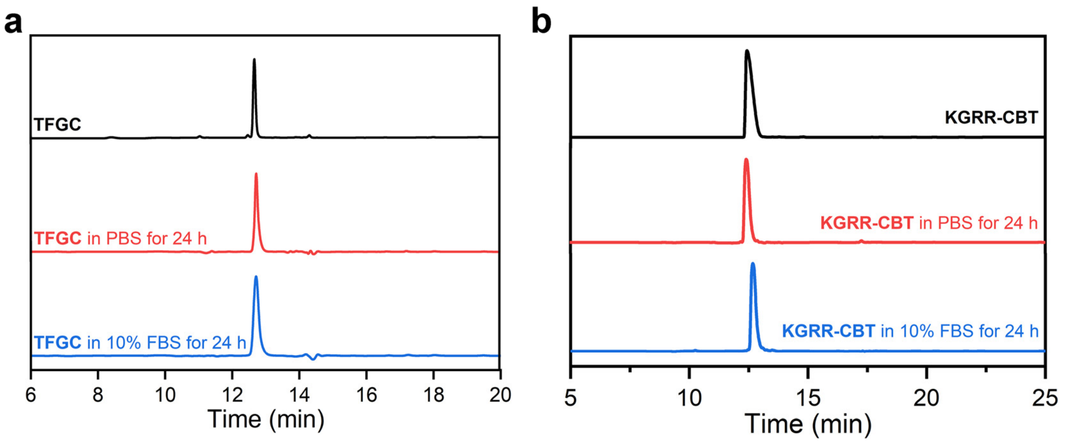

Figure S25. HPLC traces of **TFGC** (a) and **KGRR-CBT** (b) before/after incubation in PBS or 10% FBS at 37 ºC for 24 h.

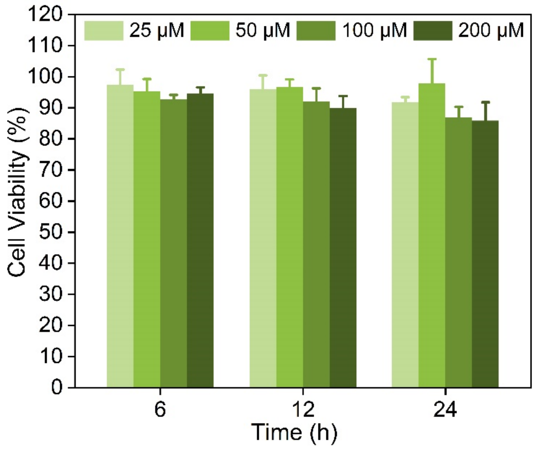

Figure S26. Cell viabilities of fLuc-transfected MDA-MB-231 cells indicated with **TFGC** at different concentrations for 6 h, 12 h, and 24 h.

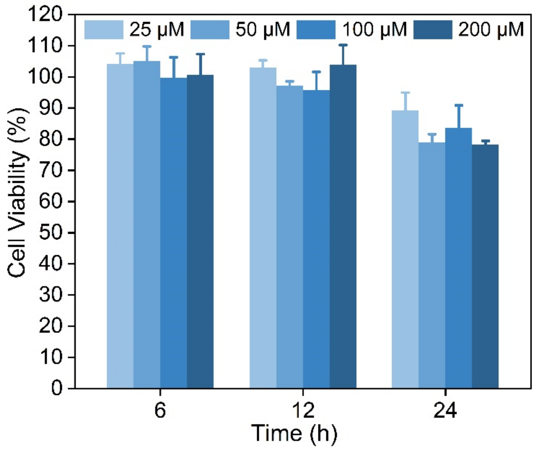

Figure S27. Cell viabilities of fLuc-transfected MDA-MB-231 cells indicated with **KGRR-CBT** at different concentrations for 6 h, 12 h, and 24 h.

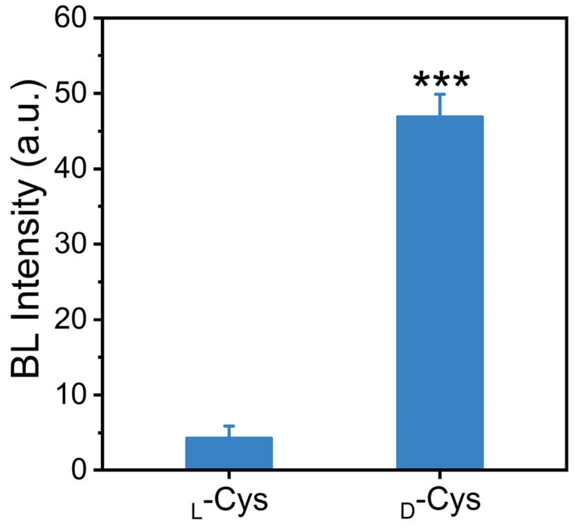

Figure S28. BL intensity of 25 μM **KGRR-CBT** treated with 50 μM L-cysteine (L-Cys) or D-Cys after incubation with 40 μg/mL CTSB.

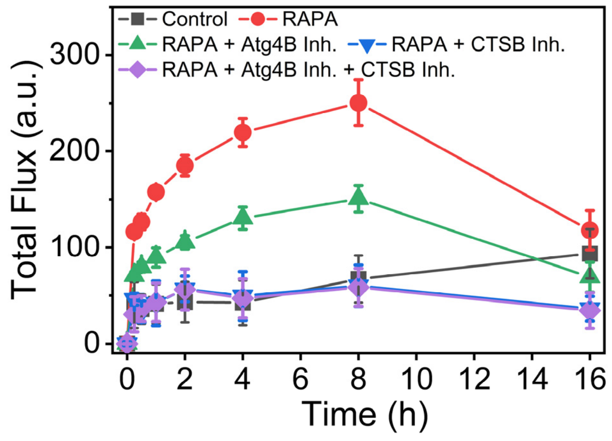

Figure S29. Time course quantified total flux of the images in Figure 2.

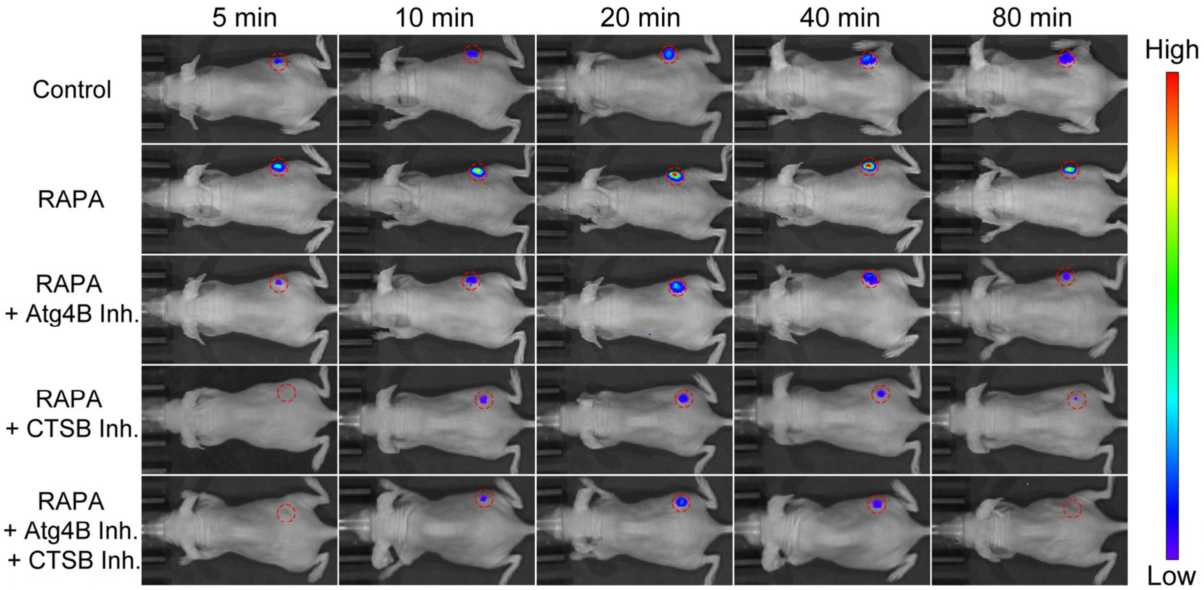

Figure S30. Time-course BL images of fLuc-transfected MDA-MB-231 tumor-bearing mice after different pretreatments followed by i.p. injection of 12.5 μmol/kg **TFGC** and 12.5 μmol/kg **KGRR-CBT**.

Table S1. The limits of detection (LODs) of Atg4B in representative reported methods.

**Probes**	**Detection Methods**	**LOD of Atg4B**	**References**
**TFGC**	bioluminescence	28.7 ng/mL	**This work**
F-pep-B HGC/Lysolite Red nanoparticle	fluorescence	not detected	2
DPBP	fluorescence	not detected	3

Table S2. The LODs of CTSB in representative reported methods.

**Probes**	**Detection Methods**	**LOD of CTSB**	**References**
**KGRR-CBT**	bioluminescence	82.6 ng/mL	**This work**
Val-Cit-Al	bioluminescence	0.027 U/L	4
DBDY-Gly-Phe-MTPA	colorimetry	5 nM	5
Gly-Arg-Arg-Gly-Lys-Gly-Gly	fluorescence	0.725 nM	6
DBDY-(Gly-INH)$_2$	fluorescence	0.5 nM	7
CB-CNP	fluorescence	not detected	8
TFB	fluorescence	not detected	9
CyA-P-CyB	fluorescence	not detected	10

Table S3. Kinetic parameters for Atg4B-catalyzed reactions.

Substrates	K_m (μM)	k_{cat} (s^{-1})	k_{cat}/K_m ($\mu M^{-1}\ s^{-1}$)	References
**Ac-TFGC**	39.98	1.28	0.032	**This work**
LC3B-115	50	-	not detected	11
LC3B-GST	5.1	0.46	0.090	12
GATE-16-GST	6.1	0.67	0.11	12
GABARAP-GST	5.8	0.46	0.079	12
Atg8L-GST	4.4	0.39	0.089	12

Table S4. Kinetic parameters for CTSB-catalyzed reactions.

Substrates	K_m (μM)	k_{cat} (s^{-1})	k_{cat}/K_m ($\mu M^{-1}\ s^{-1}$)	References
**KGRR-CBT** in this work	198.19	3.89	0.020	**This work**
Val-Cit-AL	139	9.94	0.07	4
Abz-GIVRAK(Dnp)-OH (pH 7.2)	156	2.3	0.015	13
Abz-GIVRAK(Dnp)-NH_2 (pH 7.2)	53	0.2	0.004	13
Ac-Cha-Leu-hSer(Bzl)-Arg-ACC	90.9	390	4.29	14
Ac-Cha-Leu-Glu(Bzl)-Arg-ACC	115	344	2.99	14
Z-Arg-Lys-AMC (pH 7.2)	-	-	0.023	15
Z-Glu-Lys-AMC (pH 7.2)	-	-	0.0006	15
Z-Phe-Arg-AMC (pH 7.2)	-	-	0.029	15

4. References

(1) Mofford, D. M.; Adams, S. T., Jr.; Reddy, G. S.; Reddy, G. R.; Miller, S. C., Luciferin Amides Enable in Vivo Bioluminescence Detection of Endogenous Fatty Acid Amide Hydrolase Activity. *J. Am. Chem. Soc.* **2015,** *137* (27), 8684-8687.

(2) Choi, K. M.; Nam, H. Y.; Na, J. H.; Kim, S. W.; Kim, S. Y.; Kim, K.; Kwon, I. C.; Ahn, H. J., A monitoring method for Atg4 activation in living cells using peptide-conjugated polymeric nanoparticles. *Autophagy* **2011,** *7* (9), 1052-1062.

(3) Lin, Y. X.; Qiao, S. L.; Wang, Y.; Zhang, R. X.; An, H. W.; Ma, Y.; Rajapaksha, R. P.; Qiao, Z. Y.; Wang, L.; Wang, H., An in Situ Intracellular Self-Assembly Strategy for Quantitatively and Temporally Monitoring Autophagy. *ACS Nano* **2017,** *11* (2), 1826-1839.

(4) Ni, Y.; Hai, Z.; Zhang, T.; Wang, Y.; Yang, Y.; Zhang, S.; Liang, G., Cathepsin B Turning Bioluminescence "On" for Tumor Imaging. *Anal. Chem.* **2019,** *91* (23), 14834-14837.

(5) Kim, C. J.; Lee, D. I.; Kim, C.; Lee, K.; Lee, C. H.; Ahn, I. S., Gold nanoparticles-based colorimetric assay for cathepsin B activity and the efficiency of its inhibitors. *Anal. Chem.* **2014,** *86* (8), 3825-3833.

(6) Ryu, J. H.; Kim, S. A.; Koo, H.; Yhee, J. Y.; Lee, A.; Na, J. H.; Youn, I.; Choi, K.; Kwon, I. C.; Kim, B. S.; Kim, K., Cathepsin B-sensitive nanoprobe for in vivo tumor diagnosis. *J. Mater. Chem.* **2011,** *21* (44), 17631-17634.

(7) Kim, C. J.; Lee, D. I.; Zhang, D.; Lee, C. H.; Ahn, I. S., New strategy for selective and sensitive assay of cathepsin B using a dityrosine-based material. *Anal. Biochem.* **2013,** *435* (2), 166-173.

(8) Ryu, J. H.; Na, J. H.; Ko, H. K.; You, D. G.; Park, S.; Jun, E.; Yeom, H. J.; Seo, D. H.; Park, J. H.; Jeong, S. Y.; Kim, I. S.; Kim, B. S.; Kwon, I. C.; Choi, K.; Kim, K., Non-invasive optical imaging of cathepsin B with activatable fluorogenic nanoprobes in various metastatic models. *Biomaterials* **2014,** *35* (7), 2302-2311.

(9) Lock, L. L.; Cheetham, A. G.; Zhang, P.; Cui, H., Design and Construction of Supramolecular Nanobeacons for Enzyme Detection. *ACS Nano* **2013,** *7* (6), 4924-4932.

(10) Chen, X.; Lee, D.; Yu, S.; Kim, G.; Lee, S.; Cho, Y.; Jeong, H.; Nam, K. T.; Yoon, J., In vivo near-infrared imaging and phototherapy of tumors using a cathepsin B-activated fluorescent probe. *Biomaterials* **2017,** *122*, 130-140.

(11) Tang, Y.; Kay, A.; Jiang, Z.; Arkin, M. R., LC3B Binds to the Autophagy Protease ATG4b with High Affinity Using a Bipartite Interface. *Biochemistry* **2022,** *61* (21), 2295-2302.

(12) Li, M.; Hou, Y.; Wang, J.; Chen, X.; Shao, Z. M.; Yin, X. M., Kinetics comparisons of mammalian Atg4 homologues indicate selective preferences toward diverse Atg8 substrates. *J. Biol. Chem.* **2011,** *286* (9), 7327-7338.

(13) Yoon, M. C.; Hook, V.; O'Donoghue, A. J., Cathepsin B Dipeptidyl Carboxypeptidase and Endopeptidase Activities Demonstrated across a Broad pH Range. *Biochemistry* **2022,** *61* (17), 1904-1914.

(14) Poreba, M.; Groborz, K.; Vizovisek, M.; Maruggi, M.; Turk, D.; Turk, B.; Powis, G.; Drag, M.; Salvesen, G. S., Fluorescent probes towards selective cathepsin B detection and visualization in cancer cells and patient samples. *Chem. Sci.* **2019,** *10* (36), 8461-8477.

(15) Yoon, M. C.; Solania, A.; Jiang, Z.; Christy, M. P.; Podvin, S.; Mosier, C.; Lietz, C. B.; Ito, G.; Gerwick, W. H.; Wolan, D. W.; Hook, G.; O'Donoghue, A. J.; Hook, V., Selective Neutral pH Inhibitor of Cathepsin B Designed Based on Cleavage Preferences at Cytosolic and Lysosomal pH Conditions. *ACS Chem. Biol.* **2021,** *16* (9), 1628-1643.
